# Alternative rapamycin treatment regimens mitigate the impact of rapamycin on glucose homeostasis and the immune system

**DOI:** 10.1111/acel.12405

**Published:** 2015-10-13

**Authors:** Sebastian I. Arriola Apelo, Joshua C. Neuman, Emma L. Baar, Faizan A. Syed, Nicole E. Cummings, Harpreet K. Brar, Cassidy P. Pumper, Michelle E. Kimple, Dudley W. Lamming

**Affiliations:** ^1^ Department of MedicineUniversity of Wisconsin‐MadisonMadisonWIUSA; ^2^ William S. Middleton Memorial Veterans HospitalMadisonWIUSA; ^3^ Interdisciplinary Graduate Program in Nutritional SciencesUniversity of Wisconsin‐MadisonMadisonWIUSA; ^4^ Endocrinology and Reproductive Physiology Graduate Training ProgramUniversity of Wisconsin‐MadisonMadisonWIUSA; ^5^ University of Wisconsin Carbone Cancer CenterMadisonWIUSA

**Keywords:** aging, mechanistic target of rapamycin, mice, rapamycin

## Abstract

Inhibition of the mechanistic target of rapamycin (mTOR) signaling pathway by the FDA‐approved drug rapamycin has been shown to promote lifespan and delay age‐related diseases in model organisms including mice. Unfortunately, rapamycin has potentially serious side effects in humans, including glucose intolerance and immunosuppression, which may preclude the long‐term prophylactic use of rapamycin as a therapy for age‐related diseases. While the beneficial effects of rapamycin are largely mediated by the inhibition of mTOR complex 1 (mTORC1), which is acutely sensitive to rapamycin, many of the negative side effects are mediated by the inhibition of a second mTOR‐containing complex, mTORC2, which is much less sensitive to rapamycin. We hypothesized that different rapamycin dosing schedules or the use of FDA‐approved rapamycin analogs with different pharmacokinetics might expand the therapeutic window of rapamycin by more specifically targeting mTORC1. Here, we identified an intermittent rapamycin dosing schedule with minimal effects on glucose tolerance, and we find that this schedule has a reduced impact on pyruvate tolerance, fasting glucose and insulin levels, beta cell function, and the immune system compared to daily rapamycin treatment. Further, we find that the FDA‐approved rapamycin analogs everolimus and temsirolimus efficiently inhibit mTORC1 while having a reduced impact on glucose and pyruvate tolerance. Our results suggest that many of the negative side effects of rapamycin treatment can be mitigated through intermittent dosing or the use of rapamycin analogs.

## Introduction

Rapamycin is an FDA‐approved compound that robustly extends lifespan in yeast, worms, flies, and mice (Johnson *et al*., 2013). Rapamycin is an acute inhibitor of the mechanistic target of rapamycin (mTOR) complex 1 (mTORC1), a protein kinase which regulates numerous cellular processes including ribosomal biogenesis, protein translation, and autophagy through the phosphorylation of substrates that include S6K1, 4E‐BP1, and Ulk1. Mice lacking *S6K1* or with decreased mTORC1 activity have extended longevity, demonstrating that decreased mTORC1 signaling is sufficient to promote longevity, especially in females (Selman *et al*., 2009; Lamming *et al*., 2012; Wu *et al*., 2013).

Unfortunately, the potential for serious side effects in humans, including immunosuppression and glucose intolerance, may preclude the long‐term prophylactic use of rapamycin as a therapy for age‐related diseases (Lamming *et al*., 2013b). While investigating the mechanistic basis for the effect of rapamycin on glucose tolerance, we discovered that long‐term treatment with rapamycin also inhibits a second mTOR complex, mTORC2, *in vivo*, resulting in hepatic insulin resistance (Lamming *et al*., 2012, 2013a). mTORC2 has also recently been shown to have an important role in promoting immune function, which suggests that the immunosuppressive effects of rapamycin may be due in part to the inhibition of mTORC2 signaling (Powell *et al*., 2012; Byles *et al*., 2013; Festuccia *et al*., 2014). Finally, we recently completed a lifespan study, finding that genetic inhibition of mTORC2 was severely deleterious to survival of male, but not female, mice (Lamming *et al*., 2014b).

The evidence to date suggests that the inhibition of mTORC1 will promote longevity and retard age‐related diseases, while the inhibition of mTORC2 is likely deleterious to health and impairs glucose homeostasis and the immune system. While rapamycin inhibits both complexes, it is a potent and acute inhibitor of mTORC1, and inhibits mTORC2 signaling only after prolonged treatment (Sarbassov *et al*., 2006). Interestingly, rapamycin administration for 2 weeks out of every four can significantly extend lifespan (Anisimov *et al*., 2010, 2011), demonstrating that rapamycin administration does not have to be continuous to extend lifespan. However, the impact of rapamycin on glucose homeostasis persists for 2 weeks following cessation of rapamycin (Yang *et al*., 2012; Liu *et al*., 2014), suggesting that this dosing strategy may not minimize side effects. Glucose intolerance was also observed in a recent study of mice receiving rapamycin three times per week (Leontieva *et al*., 2014).

In this study, we tested several intermittent rapamycin treatment schedules to identify the most frequent rapamycin dosing schedule that is still compatible with glucose tolerance in C57BL/6J mice. We then compared the impact of daily rapamycin treatment with the impact of the selected intermittent dosing schedule on glucose tolerance, pyruvate tolerance, and *in vivo* and *ex vivo* beta cell function, and examined the impact on T‐cell populations in splenocytes. Importantly, we find that mTORC1 inhibition is sustained in many tissues despite intermittent dosing. Finally, we compared the impact of daily rapamycin treatment on glucose homeostasis and the immune system with the impact of two FDA‐approved rapamycin analogs, everolimus and temsirolimus. Both everolimus and temsirolimus efficiently inhibited mTORC1 signaling, but had a reduced impact on glucose homeostasis compared to rapamycin.

## Results

### Intermittent treatment with rapamycin has a reduced effect on glucose tolerance

We first sought to identify a rapamycin dosing schedule that would selectively inhibit mTORC1 while minimizing deleterious, mTORC2‐mediated effects on glucose metabolism and immune system. We treated 9‐week‐old male C57BL/6J mice for 2 weeks with vehicle or 2 mg/kg rapamycin every day (1×/day) or weekly (1×/7 days), and analyzed the effect on glucose tolerance. We performed a fasting glucose tolerance test 7 days after the most recent rapamycin treatment of the weekly (1×/7 days) group; the effects of chronic rapamycin treatment on glucose tolerance persist for 2 weeks (Yang *et al*., 2012; Liu *et al*., 2014).

**Figure 1:**
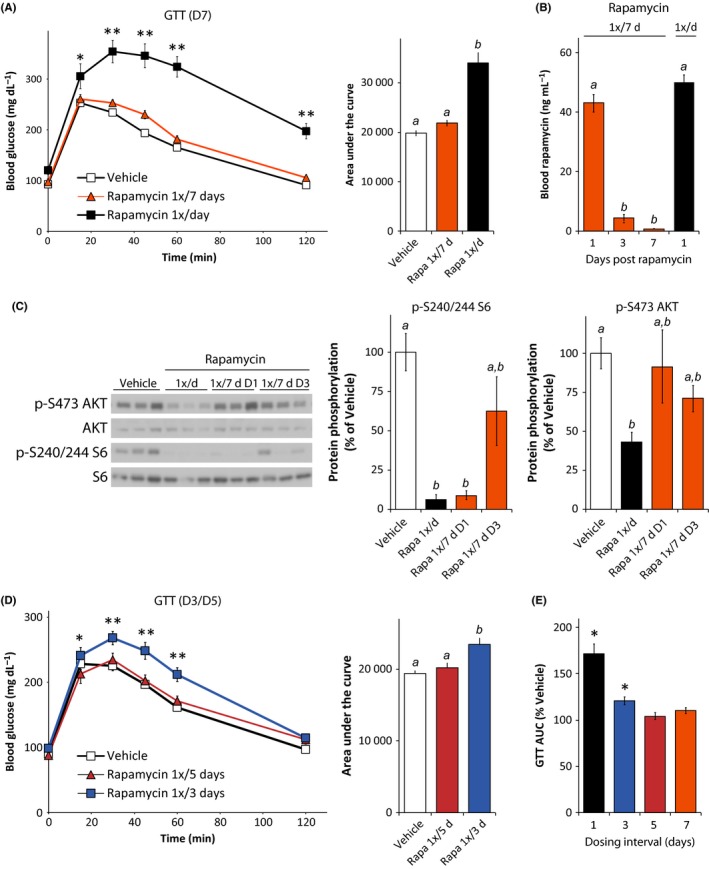
Intermittent treatment with rapamycin minimizes glucose intolerance and mTORC2 inhibition. Glucose tolerance test on male C57BL/6J mice (A) treated with vehicle or with 2 mg/kg rapamycin (1×/day or 1×/7 days) for 2 weeks, performed 7 days after the last treatment of the rapamycin 1×/7 days group (D7) [*n* = 10 vehicle, *n* = 9 1×/day rapamycin, and *n* = 11 1×/7 days rapamycin; for GTT, *P < 0.05, **P < 0.0001 vs. all groups, Tukey–Kramer test following two‐way repeated‐measures anova; for AUC, means with the same letter are not significantly different from each other (Tukey–Kramer test following one‐way anova,* P* < 0.05)]. (B) Rapamycin concentration in blood from male C57Bl/6J mice treated with 2 mg/kg rapamycin (1×/day or 1×/7 days) for 8 weeks; blood from 1×/7 days mice was collected 1 day (D1), 3 days (D3), or 7 days (D7) after the more recent rapamycin injection (*n* = 3–6/group; means with the same letter are not significantly different from each other (Tukey–Kramer test following one‐way anova,* P* < 0.05). (C) Western blotting analysis and quantification of phosphorylated S6 (S240/244) and Akt (S473) phosphorylation in skeletal muscle [*n* = 9 vehicle, 7 1×/day rapamycin, 3 rapamycin 1×/7d D1, 6 rapamycin 1×/7d D3; means with the same letter are not significantly different from each other (Tukey–Kramer test following one‐way anova,* P* < 0.05)]. (D) Glucose tolerance test on mice treated intermittently with either vehicle or with 2 mg/kg rapamycin (1×/3 or 5 days) for 2 weeks, performed 3 days after the last treatment of the rapamycin 1×/3 days group and 5 days after the last treatment of the 1×/5 days group [*n* = 11/, for GTT, *P < 0.05 rapamycin 1×/3 days vs. rapamycin 1×/5 days, **P < 0.02 rapamycin 1×/3 days vs. all groups, Tukey–Kramer test following two‐way repeated‐measures anova; for AUC, means with the same letter are not significantly different from each other (Tukey–Kramer test following one‐way anova,* P* < 0.05)]. (E) Area under the curve for the glucose tolerance tests in Fig. 1A and 1D, expressed as percent of the AUC for vehicle‐treated mice in the corresponding experiment (*P < 0.001 vs. vehicle in the corresponding experiment, two‐tailed t‐test). Error bars represent standard error.

As expected, we found that daily rapamycin treatment significantly impairs the performance of mice during a glucose tolerance test (GTT), with a 20–116% increase in blood glucose levels at every time point (Fig. 1A, left), and a 71% increase in total glucose burden over the time course of the assay as measured by area under the curve (AUC) (Fig. 1A, right). In comparison, weekly rapamycin treatment did not impair glucose tolerance, whether measured at each time point or by AUC (Fig. 1A). We observed similar results in a GTT performed after 5 weeks of treatment, on the 4th day after the most recent treatment of the weekly (1×/7 days) group (Fig. S1A). In agreement with our previous findings using diet‐delivered rapamycin (Lamming *et al*., 2013a), neither daily nor weekly rapamycin treatment increased the level of glycated hemoglobin (Fig. S1B).

In order to understand how these results related to the pharmacokinetics of rapamycin treatment, we determined the rapamycin content of blood from mice treated either daily or weekly with rapamycin for 8 weeks. Sixteen hours following administration of rapamycin, we observed similar rapamycin levels in mice treated daily with rapamycin (1×/day) and mice treated weekly (1×/7 days) (Fig. 1B, day 1). As expected, the rapamycin content of blood decreased sharply with time in the weekly treated mice, reaching the detection threshold (1 ng/mL) 7 days after the injection. We calculate that the half‐life of rapamycin in mouse blood is approximately 15 h, with blood levels of rapamycin reaching 4.9 nm 3 days after injection, a concentration capable of inhibiting mTOR signaling in tissue culture cell lines (Sarbassov *et al*., 2006). We observed very similar rapamycin kinetics in liver (Fig. S1C).

We analyzed mTOR signaling in the muscles of mice treated with vehicle, daily rapamycin, or weekly rapamycin for 2 months (Fig. 1C). As we previously reported (Lamming *et al*., 2012), daily treatment with 2 mg/kg rapamycin efficiently inhibited the phosphorylation of both S6 S240/244, a readout of mTORC1 signaling, and AKT S473, an mTORC2 substrate. Rapamycin was equally efficacious in inhibiting phosphorylation of S6 in mice treated daily with rapamycin as in mice treated weekly (1×/7d) and sacrificed on the day following treatment (D1). We observed a similar effect in liver (Fig. S1D). However, AKT S473 phosphorylation in muscle was only inhibited in mice treated daily with rapamycin (Fig. 1C). Mice treated weekly with rapamycin but sacrificed on the third day (D3) following treatment had decreased mean phosphorylation of S6 (38% decrease) and AKT (29% decrease), but these results did not reach statistical significance. By 7 days following treatment, we did not observe any inhibition of S6 phosphorylation in either liver or muscle (Fig. S1E).

We proceeded to test the effects of two more frequent rapamycin dosing schedules, rapamycin dosed once every three (1×/3d) or five (1×/5d) days, on glucose tolerance (Fig. 1D). While rapamycin delivered 1×/3 days significantly impaired glucose tolerance, rapamycin delivered 1×/5 days had no effect on glucose tolerance (Fig. 1D), or in performance during an insulin tolerance test (Fig. S1F). All of the intermittent dosing regimens had a decreased impact on AUC compared to daily rapamycin treatment (Fig. 1E). Rapamycin treatment once every 5 days (1×/5 days) had the smallest impact on glucose tolerance, and we therefore selected this dosing schedule for further analysis.

### Rapamycin treatment once every 5 days has a decreased impact on glucose homeostasis relative to daily rapamycin

**Figure 2:**
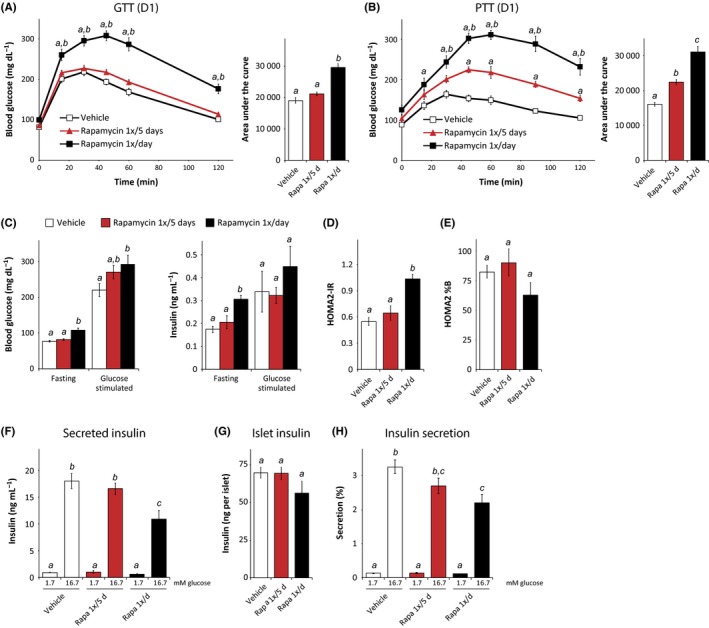
Reduced impact of intermittent rapamycin administration on glucose homeostasis. (A) Glucose and (B) pyruvate tolerance test on male C57BL/6J mice treated with vehicle or with 2 mg/kg rapamycin (1×/day or 1×/5 days) for 2 or 3 weeks, respectively [(*n* = 9 per treatment; for GTT/PTT, Tukey–Kramer test following two‐way repeated‐measures anova, a = *P* < 0.05 vs. vehicle, b = *P* < 0.05 vs. rapamycin 1×/5 days; for AUC, means with the same letter are not significantly different from each other (Tukey–Kramer test following one‐way anova,* P* < 0.05)]. (C) Fasting and glucose‐stimulated insulin secretion (GSIS) were measured by fasting mice treated for 5 weeks overnight, collecting serum, injecting 1 g/kg glucose, and collecting serum 15 min after injection [*n* = 9/group (glucose), 4/group (insulin), means with the same letter are not significantly different from each other (Tukey–Kramer test following one‐way anova,* P* < 0.05)]. (D, E) HOMA2‐IR and HOMA2%B were calculated using the fasting insulin data in C and fasting glucose data from the same mice [*n* = 4/group, means with the same letter are not significantly different from each other (Tukey–Kramer test following one‐way anova,* P* < 0.05)]. (F–H) Islets were isolated from vehicle and rapamycin (1×/day or 1×/5 days) mice treated for 8 weeks and were analyzed to determine insulin secretion in response to low (1.7 mm) and high (16.7 mm) glucose [*n* = 6 mice per treatment, means with the same letter are not significantly different from each other (Tukey–Kramer test following one‐way anova,* P* < 0.05)]. Error bars represent standard error.

We treated a new cohort of C57BL/6J males with vehicle, rapamycin dosed intermittently once every 5 days (1×/5 days), or rapamycin dosed daily (1×/day) at 2 mg/kg starting at 9 weeks of age. After 3 weeks, we performed a fasting glucose tolerance test on the day immediately following administration of rapamycin to the 1×/5 days mice. We observed no effect of intermittent rapamycin on glucose tolerance, while daily rapamycin treatment caused a robust decrease in glucose tolerance (Fig. 2A). One treatment cycle later, we performed a pyruvate tolerance test (PTT); pyruvate can be utilized as a substrate for gluconeogenesis by the liver, permitting us to assess hepatic gluconeogenesis (Houde *et al*., 2010; Lamming *et al*., 2012). As expected, daily rapamycin treatment induced significant pyruvate intolerance (Fig. 2B), indicating increased levels of hepatic gluconeogenesis. Mice treated intermittently with rapamycin were also pyruvate intolerant, although the increase in the AUC induced by daily rapamycin treatment was twice that observed in the AUC of mice treated intermittently (Fig. 2B).

Chronic rapamycin results in fasting hyperglycemia in both humans and mice (Lamming *et al*., 2013b). After 5 weeks of treatment, mice treated daily with rapamycin, but not mice treated intermittently, showed fasting hyperglycemia and hyperinsulinemia (Fig. 2C). We used these measures to calculate insulin resistance and beta cell sensitivity using homeostasis model assessment (HOMA2‐IR) (Levy *et al*., 1998). The HOMA2‐IR model was derived empirically from human insulin–glucose clamp data, but remains a useful surrogate measure of insulin resistance in mice (Mather, 2009). Mice treated daily with rapamycin had a significantly higher HOMA2‐IR value than mice treated intermittently with rapamycin or mice treated with vehicle (Fig. 2D).

We observed a trend toward decreased beta cell function (HOMA2%B, a surrogate measurement of beta cell function) in mice treated daily with rapamycin (Fig. 2E), although this was not statistically significant. With significant evidence that rapamycin impairs beta cell function (Barlow *et al*., 2013), we decided to investigate the impact of rapamycin dosing on islet function more closely. We isolated pancreatic islets from mice after 8 weeks of rapamycin treatment and performed an *ex vivo* glucose‐stimulated insulin secretion assay (Kimple *et al*., 2013) (Fig. 2F–H). Islets from mice treated daily with rapamycin secreted significantly less insulin into the media than mice treated with vehicle or intermittently treated with rapamycin (Fig. 2F), and we also observed a slight reduction in insulin content in these islets (Fig. 2G). When we calculated the percentage of insulin secreted, we found that islets from mice treated daily with rapamycin were significantly less responsive to glucose stimulation than islets from vehicle‐treated mice (Fig. 2H). Intermittent treatment with rapamycin had little to no effect on secreted insulin, islet insulin content, or the percent insulin secreted in response to glucose stimulation, demonstrating that beta cell function is maintained in mice treated intermittently with rapamycin.

### Impact of intermittent rapamycin treatment on mTOR signaling and testes weight

**Figure 3:**
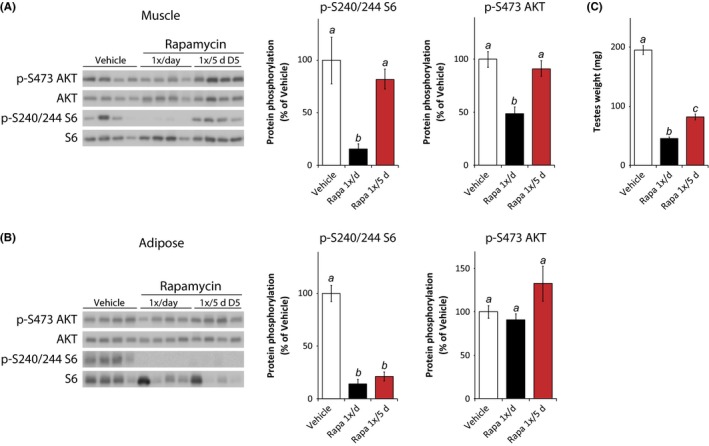
Sustained impact of intermittent rapamycin on adipose mTORC1 and testes weight. (A) Muscle lysate and (B) adipose tissue lysate were analyzed by Western blotting, and the phosphorylation of S6 240/244 and AKT S473 relative to their respective total protein was quantified. Tissues were collected from mice treated with vehicle or rapamycin (1×/day or 1×/5 days) for 8 weeks, with the tissue collection scheduled such that the intermittent rapamycin treatment group was sacrificed 5 days after the previous rapamycin injection. Mice were fasted overnight and sacrificed following stimulation with 0.75 U/kg insulin for 15 min. Islets were isolated as described prior to tissue collection [*n* = 5–9 per treatment, means with the same letter are not significantly different from each other (Tukey–Kramer test following one‐way anova,* P* < 0.05)]. (C) The testes of mice in each treatment group were weighed [*n* = 9 per group, means with the same letter are not significantly different from each other (Tukey–Kramer test following one‐way anova,* P* < 0.05)]. Error bars represent standard error.

We analyzed mTOR signaling in several tissues from the same mice used for *ex vivo* islet analysis. After 8 weeks of receiving vehicle, intermittent (1×/5 days) rapamycin, or daily rapamycin, mice were sacrificed on ‘day 5’ (D5)—5 days following the last administration of rapamycin to the intermittent treatment group—in order to determine whether mTOR inhibition persists between treatments. We observed a significant decrease in the phosphorylation of S6 in the muscle, liver, heart, and pancreatic islets of mice receiving daily rapamycin, but no change in S6 phosphorylation in the D5 intermittent treatment group (Fig. 3A and Fig. S2A–C), although there was a clear trend toward reduced S6 phosphorylation in pancreatic islets isolated from the D5 intermittent treatment group (Fig. S2C). Similarly, we observed decreased AKT S473 phosphorylation in muscle of the daily rapamycin treatment group, but not the intermittent treatment group (Fig. 3A). Adipose tissue was unique in that we observed a sustained effect of intermittent rapamycin on S6 phosphorylation even on ‘day 5’ that was equivalent to that observed in daily rapamycin (Fig. 3B).

Analysis of mTOR signaling in harvested tissue is informative, but ideally we would be able to measure average mTOR activity over time rather than the precise activity at a single time point. A well‐known side effect of rapamycin treatment in humans and mice is testicular degeneration (Wilkinson *et al*., 2012), and we observed that both the intermittent rapamycin and daily rapamycin treatment regimens significantly decreased testes mass (Fig. 3C). Daily rapamycin decreased testes weight by approximately 75%, while the intermittent (1×/5 days) rapamycin regimen decreased testes weight by almost 60%.

### Rapamycin delivered once every 5 days has a reduced impact on the immune system

The immunosuppressive effects of rapamycin are a potential barrier to widespread use of rapamycin as a therapy for age‐related diseases (Lamming *et al*., 2013b), although the dosing strategy utilized may be of major importance. The true impact of rapamycin on the immune system is still unclear, and rapamycin may not be generally immunosuppressive—indeed, rapamycin treatment improves survival in mouse models of infection (Hinojosa *et al*., 2012; Hasty *et al*., 2014) and improves the response to vaccines in both nonhuman primates (Turner *et al*., 2011) and elderly humans (Mannick *et al*., 2014). However, in addition to a reported increase in viral and fungal infections in humans taking rapamycin (Mahe *et al*., 2005), even short‐term low‐dose rapamycin decreases defense against bacterial and viral pathogens in mice (Goldberg *et al*., 2014). While not attempting to resolve this important question, we decided to compare the impact of daily rapamycin to our intermittent treatment regimen.

**Figure 4:**
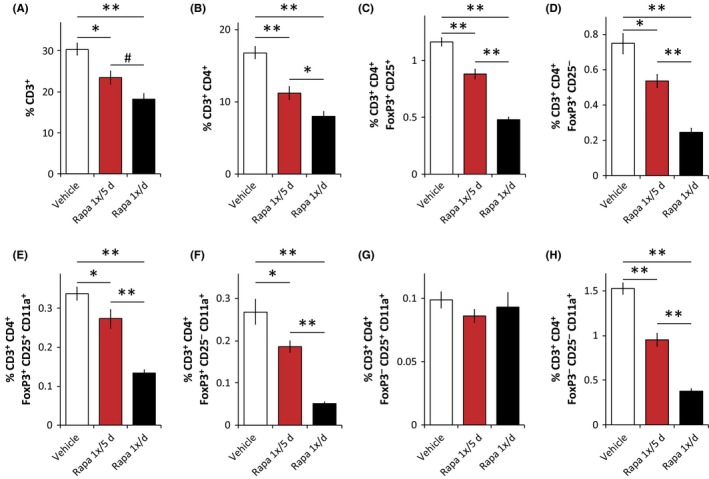
Intermittent rapamycin treatment has a reduced but significant impact on the immune system. (A–H) Flow cytometry analysis (expressed as percent of total live cells) of splenocytes from male C57BL/6J mice treated with vehicle or rapamycin (1×/day or 1×/5 days) for 8 weeks (*n* = 6–8 mice/group, ^#^ *P* < 0.052,*P < 0.05, **P < 0.0005, Tukey–Kramer test following one‐way anova). Error bars represent standard error.

We isolated splenocytes from the mice during the tissue and islet harvesting experiments described above and analyzed the splenocyte population using flow cytometry. Both the intermittent and daily rapamycin treatment regimens had a significant impact upon immune cell numbers, with daily rapamycin treatment impacting T‐cell numbers more than the intermittent rapamycin treatment regimen (Fig. 4A). This effect was observed on both CD3^+^CD4^+^ cells (Fig. 4B) and CD3^+^CD8^+^ T cells (Fig. S3A). It has previously been reported that rapamycin treatment of mice results in a decrease in blood T regulatory cells (Tregs), defined as CD3^+^CD4^+^CD25^+^Foxp3^+^ (Makki *et al*., 2014), and we observed a similar effect of daily rapamycin treatment upon Tregs isolated from the spleen (Fig. 4C). Interesting, while daily rapamycin induces an almost 60% decrease in Tregs, intermittent rapamycin treatment resulted in only a 25% decrease (Fig. 4C). Similarly, intermittent rapamycin had reduced impact on the frequency of CD25^−^ Tregs (Fig. 4D), which may constitute a reservoir of committed regulatory cells (Zelenay *et al*., 2005) and have been shown to have similar biological function to CD25^+^ Tregs (Fontenot *et al*., 2005). We observed a similar greater impact of daily rapamycin treatment on CD8^+^ cells expressing Foxp3 (Fig. S3B,C), which also function as regulatory cells (Tang *et al*., 2005).

mTORC2‐deficient T cells have decreased activation‐induced binding to ICAM‐1, a key step in the immune response (Lee *et al*., 2010). The interaction of T cells with ICAM‐1 is mediated by the integrin lymphocyte function‐associated antigen 1 (LFA1), which consists of two subunits, Cd11a and Cd11b. We therefore examined the relative impact of intermittent and daily rapamycin on the expression of Cd11a by CD25^+^ Tregs (Fig. 4E), CD25^−^ Tregs (Fig. 4F), and cells lacking Foxp3 expression (Fig. 4G‐H). In both CD25^+^ and CD25^−^ Tregs, and also in CD3^+^CD4^+^CD25^−^Foxp3^−^ cells, daily rapamycin treatment reduced the number of Cd11a^+^ T cells by 60–80%, with the intermittent rapamycin treatment regimen having a significantly reduced impact.

### Daily everolimus or temsirolimus treatment has a reduced impact on glucose and pyruvate tolerance compared to daily sirolimus

**Figure 5:**
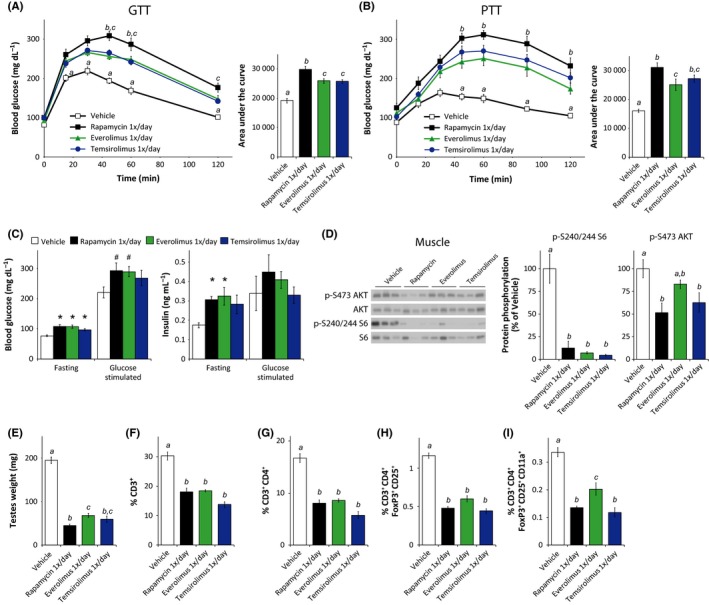
Rapamycin analogs efficiently inhibit mTORC1 but show a reduced impact on glucose homeostasis and the immune system. (A) Glucose and (B) pyruvate tolerance tests on male C57BL/6J mice treated with vehicle or with 2 mg/kg rapamycin (1×/day) or equimolar amounts of everolimus or temsirolimus for 2 or 3 weeks, respectively [*n* = 9 per treatment; for GTT/PTT, Tukey–Kramer test following two‐way repeated‐measures anova, a = *P* < 0.05 vehicle vs. all groups, b = *P* < 0.05 rapamycin vs. everolimus, c = *P* < 0.05 rapamycin vs. temsirolimus. For AUC, means with the same letter are not significantly different from each other (Tukey–Kramer test following one‐way anova,* P* < 0.05)]. (C) Fasting and glucose‐stimulated insulin secretion (GSIS) were measured by fasting mice treated for 5 weeks overnight, collecting serum, injecting 1 g/kg glucose, and collecting serum 15 min after injection. [*n* = 9/group (glucose), 4/group (insulin), # *P* ≤ 0.08 vs. vehicle, *P ≤ 0.05 vs. vehicle, Dunnett's test following one‐way anova)]. (D) Muscle lysate was analyzed by Western blotting and the phosphorylation of S6 240/244 and AKT S473 relative to their respective total protein was quantified [*n* = 5–6 per treatment; means with the same letter are not significantly different from each other (Tukey–Kramer test following one‐way anova,* P* < 0.05)]. (E) The testes of mice in each treatment group were weighed [*n* = 9/group, means with the same letter are not significantly different from each other (Tukey–Kramer test following one‐way anova,* P* < 0.05)]. (F–I) Flow cytometry analysis (expressed as percent of total live cells) on splenocytes isolated from each treatment group [*n* = 3–8 mice/group, means with the same letter are not significantly different from each other (Tukey–Kramer test following one‐way anova,* P* < 0.05)]. The experiments presented here were conducted in parallel with the experiment presented in Figs 2, 3, 4, and the vehicle and daily (rapamycin 1×/day) data are duplicated here for ease of comparison. Error bars represent standard error.

Since the initial discovery of rapamycin, several rapamycin analogs (rapalogs) have been developed to improve the pharmacokinetics of rapamycin (sirolimus/Rapamune). The most widely used are everolimus and temsirolimus, which are both FDA‐approved for specific types of cancer and are in numerous clinical trials. While comparing side effects across clinical trials is difficult, it has been suggested that the side effect profiles of rapalogs in humans may differ (Sankhala *et al*., 2009). We therefore decided to compare the effect of daily dosing of 2 mg/kg rapamycin (sirolimus/Rapamune) to daily dosing of equimolar quantities of everolimus and temsirolimus (Figure 5). These experiments were conducted in parallel with the experiments conducted in Figures 2, 3, 4, and the vehicle and daily (rapamycin 1×/day) data are duplicated for ease of comparison.

We performed glucose (Fig. 5A) and pyruvate (Fig. 5B) tolerance tests on everolimus‐ and temsirolimus‐treated mice and assessed fasting and glucose‐stimulated glucose and insulin levels (Fig. 5C). Despite the very similar effects of daily rapamycin, everolimus, and temsirolimus on fasting blood glucose (Fig. 5C), everolimus and temsirolimus both had a reduced impact on glucose tolerance compared to daily rapamycin (Fig. 5A). A similar effect was observed with respect to pyruvate tolerance, with everolimus having 40% less impact than rapamycin on AUC (Fig. 5B). With regard to fasting and glucose‐stimulated glucose and insulin levels, daily treatment with all three rapalogs showed similar effects (Fig. 5C). We likewise calculated HOMA2‐IR and found similar effects of all three rapalogs on HOMA2‐IR, %S, and %B (Fig. S4A–C).

We wondered whether the reduced effect of the rapamycin analogs on glucose and pyruvate tolerance was correlated with reduced inhibition of mTOR signaling, and we analyzed the impact of the three rapalogs on S6 and AKT S473 phosphorylation. In muscle, liver, adipose, and heart tissue, we found that all three rapalogs were equally efficacious in inhibiting S6 phosphorylation (Fig. 5D and S5A–C). Interestingly, while daily rapamycin and temsirolimus inhibited AKT S473 phosphorylation in muscle (Fig. 5D), everolimus had no significant effect. All three rapalogs significantly reduced testes weight, although the testes of everolimus‐treated mice were slightly heavier than those from rapamycin‐treated mice (Fig. 5E), and were equivalent in weight to testes from mice treated 1×/5 days with rapamycin (Fig. 3C).

Finally, we analyzed the relative impact on immune cells of the three rapalogs. All three rapalogs showed a significant impact on the T‐cell populations analyzed, including total CD3^+^ cell (Fig. 5F), CD3^+^CD4^+^ cells (Fig. 5G), Tregs (Fig. 5H), and Tregs expressing CD11a (Fig. 5I). Similar effects were observed on the CD8^+^ population, including those expressing Foxp3, and on other CD3^+^CD4^+^ cells populations expressing Cd11a (Fig. S6). Everolimus showed a slightly reduced impact on Tregs expressing Cd11a relative to both sirolimus and temsirolimus (Fig. 5I).

## Discussion

The extensive side effect profile of rapamycin in humans may be a significant challenge to the potential use of rapamycin for age‐related diseases. This is especially true if rapamycin needs to be taken continuously as a prophylactic measure. If rapamycin could be used intermittently, some of these effects might be avoided. Several experiments have explored the use of ‘intermittent’ rapamycin; while these experiments have increased mice lifespan, the dosing schedules used (e.g., 2 weeks on rapamycin followed by a 2‐week drug holiday) are, based on previous work from many laboratories and our present results, long enough to significantly impair metabolism and immunity throughout the treatment interval as well as during the drug holiday period (Anisimov *et al*., 2010, 2011).

Here, we instead utilized an intermittent treatment regimen with single doses of rapamycin separated by a relatively short time interval that we have experimentally determined minimizes the effect of rapamycin on glucose tolerance. We found that rapamycin remains in the blood at detectable levels for at least 3 days and that dosing mice with rapamycin once every 5 days has no significant impact on glucose tolerance (Fig. 1). In contrast to daily rapamycin administration, which caused fasting hyperglycemia and hyperinsulinemia and impaired glucose‐stimulated insulin secretion (GSIS) from beta cells, intermittent (1×/5 days) rapamycin treatment did not impact fasting glucose and insulin levels, and had a minimal impact on GSIS (Fig. 2). A reduced but still significant impact of intermittent rapamycin on T cells was also observed (Fig. 4). As mTORC2 in T cells may be extremely sensitive to rapamycin (Powell *et al*., 2012), it is not clear whether this remaining impact on the immune system is primarily the result of mTORC1 inhibition, or also reflects residual inhibition of mTORC2.

Surprisingly, while intermittent rapamycin administration had no impact on glucose tolerance (Fig. 2A), it had a significant effect on pyruvate tolerance (Fig. 2B), indicating increased hepatic gluconeogenesis. The different outcomes of these two tests were unexpected, as we have previously observed that chronic rapamycin treatment induces both glucose and pyruvate intolerance due to increased hepatic gluconeogenesis and hepatic insulin resistance (Lamming *et al*., 2012, 2013a). Rictor is an essential protein component of mTORC2, and we showed that the effects of rapamycin on hepatic insulin resistance are mediated by disruption of mTORC2 utilizing a whole‐body tamoxifen‐inducible *Rictor* knockout mouse (Lamming *et al*., 2012).

As *Rictor* was deleted throughout the body of the mice in this study, the site (or sites) of rapamycin action on mTORC2 that mediates these glycemic phenotypes is not clear. Although tissue‐specific deletion of *Rictor* in liver is sufficient to cause hepatic insulin resistance, this is also a feature of mice lacking *Rictor* in adipose tissue, and mice lacking *Rictor* in skeletal muscle or pancreatic beta cells also have impaired glucose tolerance (Kumar *et al*., 2008, 2010; Gu *et al*., 2011; Lamming *et al*., 2012, 2014a). Inhibition of mTORC2 in multiple tissues may contribute to the organismal phenotype of mice chronically treated with rapamycin, and the distinct effects of chronic and intermittent rapamycin on glucose tolerance may be due to chronic and intermittent rapamycin impairing mTORC2 in distinct sets of tissues. A full mechanistic explanation of this effect will likely require the development of additional mouse models.

Consistent with its effects on glucose homeostasis, chronic rapamycin treatment can disrupt mTORC2 signaling in liver, skeletal muscle, and adipose tissues as well as many others (Lamming *et al*., 2012; Schreiber *et al*., 2015). Interestingly, in the present study, we observe decreased AKT S473 phosphorylation in the skeletal muscle of mice treated daily with rapamycin (Fig. 3A), but not in adipose tissue (Fig. 3B) or liver (Fig. S2A). The effect of chronic rapamycin treatment on AKT S473 phosphorylation can be difficult to observe and is time sensitive (Schreiber *et al*., 2015), and so we may have only observed this effect in skeletal muscle due to the time point utilized. The impact of chronic rapamycin on AKT S473 phosphorylation is also influenced by diet (Liu *et al*., 2014). The current study utilized a different chow (LabDiet 5001) than we utilized in our previous studies (ProLab RMH 3000), yet the phenotypes of rapamycin‐treated mice were identical. One possible interpretation of our current results is that the inhibition of mTORC2 in skeletal muscle and/or specific other extra‐hepatic tissues is sufficient to inhibit hepatic insulin sensitivity. Alternatively, the effect of chronic rapamycin treatment in the liver may be independent of AKT, and mediated by other hepatic substrates of mTORC2 such as SGK (Lamming *et al*., 2014a). Further studies will be required to distinguish between these distinct possibilities.

As we anticipated following our preliminary experiments, our intermittent rapamycin regimen does not continuously inhibit mTORC1 throughout the 5‐day period in mouse tissues (Fig. 3). Fascinatingly, in adipose tissue (Fig. 3B), we observed that S6 phosphorylation was strongly inhibited even on day 5 (D5). We theorize that as rapamycin is highly lipophilic, its residence in fat tissue may be prolonged. The prolonged inhibition of mTORC1 signaling in adipose tissue may have significant implications for the treatment of obesity and diabetes, and may partially explain a recent report demonstrating beneficial effects of weekly rapamycin administration to mice on a high‐fat diet (Leontieva *et al*., 2014).

We measured testes weight as a convenient method of assessing the time‐integrated impact of rapamycin on mTOR signaling. Daily rapamycin reduced testes weight by almost 80%, while intermittent dosing of rapamycin reduced testes weight by almost 60% (Fig. 3C). While there is a clear difference between the effects of the two rapamycin dosing schedules on testes weight, these dramatic results demonstrate the sustained biological impact of intermittent rapamycin. Testes weight is closely correlated with sperm production in mammals (Moller, 1989), and the recently demonstrated requirement for mTORC1 activity in spermatogenesis (Baker *et al*., 2014) leads us to hypothesize that the effects of intermittent rapamycin treatment on testes weight may primarily result from mTORC1 inhibition.

Several different rapamycin analogs (rapalogs) have been approved by the FDA. These rapalogs, which include everolimus and temsirolimus, were developed in part to improve the pharmacokinetics of sirolimus. For example, everolimus has an approximately 50% shorter blood half‐life than sirolimus in humans (Formica *et al*., 2004), while *in vitro* sirolimus has a slightly lower IC_50_ against mTORC1 (Lamming *et al*., 2013b). While comparing side effects across clinical trials is difficult, it has been suggested that the side effect profiles of rapalogs in humans may differ (Sankhala *et al*., 2009). We hypothesized that the altered pharmacokinetics of everolimus and temsirolimus might lead to reduced inhibition of mTORC2 and therefore a reduction in undesirable side effects.

Notably, we observed a significantly decreased impact of everolimus and temsirolimus on both glucose and pyruvate tolerance, despite the similar effects of all three rapalogs on blood glucose and insulin levels (Fig. 5). Importantly, all three compounds were equally efficacious in inhibiting mTORC1 activity in all of the tissues examined (Figs. 5D and S5). Everolimus showed a slightly decreased impact compared to rapamycin with regard to splenocyte Cd11a^+^ Tregs and testes weight. We conclude that at least in mice, everolimus and temsirolimus efficiently inhibit mTORC1 while having a reduced impact on glucose homeostasis relative to rapamycin. The impact of this difference on the metabolic effects of rapalogs in humans remains to be determined. While the efficacy of everolimus and temsirolimus at extending lifespan and healthspan has not yet been tested, our prediction from this data is that it will be at least as effective, and possibly more so, than sirolimus. It is perhaps fortuitous that the first human trials of rapalogs for age‐related conditions have utilized everolimus (Mannick *et al*., 2014).

Our research here has investigated the possibility that the therapeutic window of rapamycin for age‐related diseases could be expanded through the use of either an intermittent rapamycin dosing schedule or FDA‐approved rapamycin analogs. With regard to the limited set of side effects examined here—notably the adverse effects on glucose homeostasis and immune cell population—we have shown that intermittent administration of rapamycin or the daily administration of rapalogs can efficiently inhibit mTORC1 signaling with reduced negative side effects compared to daily administration of rapamycin. While important unanswered questions remain, including the impact of these strategies on other rapamycin‐associated side effects and the ability of these strategies to delay age‐related diseases, our results suggest that a carefully designed dosing strategy, possibly using a rapalog such as everolimus or temsirolimus, may enable the translation of rapamycin‐based therapies to the clinic while minimizing side effects.

## Experimental procedures

### Materials

For Western blotting, antibodies to phospho‐Akt S473 (4060), Akt (4691), phospho‐S6 ribosomal protein (2215), and S6 ribosomal protein (2217) were from Cell Signaling Technology. For flow cytometery, antibodies to CD4 (75‐0041‐U025) and CD8 (80‐0081‐U025) were from Tonbo, antibodies to CD25 (47‐0251‐80) and Foxp3 (25‐5773‐82) were from eBioscience, and antibodies to CD11a (562809) and CD3 (562332) were from BD. Protease and phosphatase inhibitor cocktail tablets were from Fisher. Other chemicals were purchased from Sigma unless noted. Glucose measurements were performed using a Bayer Contour blood glucose meter and test strips. Mouse HbA1c and Mouse insulin ELISA kits were purchased from Crystal Chem, Downers Grove, IL. Rapamycin (sirolimus), everolimus, and temsirolimus were purchased from LC Laboratories. Monoclonal insulin/proinsulin (10R‐I136a) and biotin‐conjugated (61R‐I136bBT) antibodies for islet ELISAs were purchased from Fitzgerald.

### Immunoblotting

Cells and tissue samples were lysed in cold RIPA buffer supplemented with phosphatase inhibitor and protease inhibitor cocktail tablets. Tissues were lysed in RIPA buffer as previously described (Lamming *et al*., 2012) using a FastPrep 24 (M.P. Biomedicals) with bead‐beating tubes and ceramic beads (Mo‐Bio Laboratories, Carlsbad, CA), and then centrifuged. Protein concentration was determined by Bradford (Pierce Biotechnology, Rockford, IL). Twenty microgram of protein was separated by sodium dodecylsulfate–polyacrylamide gel electrophoresis (SDS‐PAGE) on 10% resolving gels (Thermo Fisher Scientific, Waltham, MA). Imaging was performed using a GE ImageQuant LAS 4000 imaging station. Quantification was performed by densitometry using NIH ImageJ software.

### Animals and treatments

Animal studies were approved by the Institutional Animal Care and Use Committee of the University of Wisconsin‐Madison and the William S. Middleton Memorial Veterans Hospital, Madison WI. C57BL/6J mice were purchased from The Jackson Laboratory at 8–9 weeks of age, and rapamycin/rapamycin analog treatment was begun at 10 weeks of age. Glucose, insulin, and pyruvate tolerance tests were performed by fasting the mice overnight for 16 h and then injecting either glucose (1 g/kg), insulin (0.75 U/kg), or pyruvate (2 g/kg) intraperitoneally. Glucose measurements were performed using a Bayer Contour blood glucose meter and test strips.

Rapamycin (2 mg/kg) and equimolar quantities of rapamycin analogs (2.1 mg/kg everolimus and 2.25 mg/kg temsirolimus) were dissolved in ethanol and diluted in vehicle (5% Tween‐80, 5% PEG‐40) prior to intraperitoneal injection. Mice were typically injected between 3 and 5 pm, and prior to tolerance tests, any injections were performed immediately prior to commencement of the overnight fast.

Nomenclature for intermittent treatments with rapamycin: Day 1 (D1) refers to mice analyzed or sacrificed which were treated with rapamycin the previous afternoon, and Day 3 (D3), to mice analyzed or sacrificed 3 days after the most recent rapamycin treatment; similarly for Day 5 (D5) and Day 7 (D7). All mice were analyzed or sacrificed between 8 am and 12 pm.

### Rapamycin quantification, islet isolation and *ex vivo* glucose‐stimulated insulin secretion (GSIS) assay, and splenocyte preparation

See supplemental methods.

### Statistics

Statistical analysis was conducted using prism 6 (GraphPad Software, San Diego, CA). Glucose, insulin, and pyruvate tolerance tests were analyzed with two‐way repeated‐measures anova followed by a Tukey–Kramer post hoc test. Area under the curves calculated from tolerance tests, and all other comparisons of three or more means, was analyzed by one‐way anova followed by a Dunnett's or Tukey–Kramer post hoc test as appropriate.

## Funding

The Lamming lab is supported by a K99/R00 Pathway to Independence Award to D.W.L. from the National Institute of Health/National Institute on Aging (AG041765), as well as startup funds from the UW‐Madison School of Medicine and Public Health and the UW‐Madison Department of Medicine. This work was also supported by grants from the American Diabetes Association (1‐14‐BS‐115) and the NIH/NIDDK (R01 DK102598) to M.E.K. D.W.L. is a member of the UW‐Carbone Cancer Center (UWCCC), and use of the UWCCC Flow Cytometry Laboratory, a Shared Service of the UWCCC, was supported in part by the University of Wisconsin Carbone Cancer Center Support Grant P30 CA014520. J.C.N. is supported in part by a training grant from the UW Institute on Aging (NIA T32 AG000213). This work was supported using facilities and resources at the William S. Middleton Memorial Veterans Hospital. This work does not represent the views of the Department of Veterans Affairs or the United States Government.

## Conflict of interest

None declared.

## Supporting information


**Figure S1** Impact of weekly rapamycin on glucose homeostasis and mTORC1 activity in the liver. A) Glucose tolerance test on male C57BL/6J mice treated with vehicle or with 2 mg/kg rapamycin (1×/day or 1×/7 days) for 5 weeks, tested 4 days (D4) after the most recent injection of the Rapamycin 1×/7 days group [*n* = 10 vehicle, *n* = 9 Rapamycin 1×/day, and *n* = 11 Rapamycin 1×/7 days rapamycin; for GTT, *P < 0.05, **P < 0.0001 vs. all groups, Tukey‐Kramer test following two‐way repeated‐measures anova; for AUC, means with the same letter are not significantly different from each other (Tukey–Kramer test following one‐way anova, p < 0.05)]. B) HbA1c was assayed from the whole blood of mice treated with vehicle or with 2 mg/kg rapamycin (1×/day or 1×/7 days). C) Rapamycin concentration in liver from male C57Bl/6J mice treated with 2 mg/kg rapamycin (1×/day or 1×/7 days) for 8 weeks; blood from 1×/7 days mice was collected 1 day (D1), 3 days (D3) or 7 days (D7) after the more recent rapamycin injection (*n* = 3‐6/group; a  =  *P* < 0.05 vs. 1×/7 days D1; b  =  *P* < 0.05 vs. 1×/day daily rapamycin mice; c = *P* < 0.05 vs. 1×/7 days D3; two‐tailed *t*‐test). (D) Western blotting analysis and quantification of phosphorylated S6 (Ser 240/244) in liver [*n* = 9 vehicle, 7 1×/day rapamycin, 3 rapamycin 1×/7days D1, 6 rapamycin 1×/7days D3; means with the same letter are not significantly different from each other (Tukey–Kramer test following one‐way anova,* P* < 0.05)]. (E) Western blotting analysis and quantification of phosphorylated S6 (Ser 240/244) in liver and muscle [*n* = 7 per group, means with the same letter are not significantly different from each other (Tukey–Kramer test following one‐way anova,* P* < 0.05)]. F) Insulin tolerance test on mice treated intermittently with either vehicle or with 2 mg/kg rapamycin (1×/3 or 5 days) for 2 weeks [*n* = 10 mice/group, * = *P* < 0.05, Tukey‐Kramer test following two‐way repeated‐measures anova; for AUC, means with the same letter are not significantly different from each other (Tukey–Kramer test following one‐way anova,* P* < 0.05)]. Error bars represent standard error.

**Figure S2** Impact of intermittent rapamycin on liver and heart. A) Liver lysate, B) Heart tissue lysate and C) Pancreatic islet lysate was analyzed by western blotting and the phosphorylation of S6 240/244 and AKT S473 relative to their respective total protein was quantified. Tissues were collected from mice treated with vehicle or rapamycin (1×/day or 1×/5 days) for 8 weeks, with the tissue collection scheduled such that the intermittent rapamycin treatment group was sacrificed 5 days after the previous rapamycin injection. Mice were fasted overnight and sacrificed following stimulation with 0.75 U/kg insulin for 15 min. Islets were isolated as described prior to tissue collection [*n* = 4–9/group, means with the same letter are not significantly different from each other (Tukey–Kramer test following one‐way anova,* P* < 0.05)]. Error bars represent standard error.

**Figure S3** Intermittent rapamycin treatment has a reduced but significant impact on the immune system. A‐H) Flow cytometry analysis (expressed as percent of total live cells) of CD8^+^ splenocytes from male C57BL/6J mice treated with vehicle or rapamycin (1×/day or 1×/5 days) for 8 weeks (*n* = 6–8 mice/group, means with the same letter are not significantly different from each other (Tukey–Kramer test following one‐way anova,* P* < 0.05). Error bars represent standard error.

**Figure S4** Impact of rapalogs on insulin resistance. A‐C) HOMA2‐IR and HOMA2%B was calculated using the fasting insulin data in Figure 5C and fasting glucose data from the same mice (*n* = 4/group, # = *P* ≤ 0.08 vs. vehicle, * = *P* ≤ 0.05 vs. vehicle, Dunnett's test following one‐way anova). The experiments presented here were conducted in parallel with the experiment presented in Figure 2, and the vehicle and daily (Rapamycin 1×/day) data is duplicated here for ease of comparison. Error bars represent standard error.

**Figure S5** Impact of rapamycin analogs on mTOR signaling in liver, adipose and heart. A‐C) Liver, adipose and heart lysate was analyzed by western blotting and the phosphorylation of S6 240/244 and AKT S473 relative to their respective total protein was quantified [*n* = 4–9/group, means with the same letter are not significantly different from each other (Tukey–Kramer test following one‐way anova,* P* < 0.05)]. Error bars represent standard error.

**Figure S6** Impact of rapamycin analogs on splenocyte populations. Flow cytometry analysis (expressed as percent of total live cells) on splenocytes isolated from each treatment group [*n* = 3–8 mice/group, means with the same letter are not significantly different from each other (Tukey–Kramer test following one‐way anova,* P* < 0.05)]. The experiments presented here were conducted in parallel with the experiment presented in Figures 4 and S3, and the vehicle and daily (Rapamycin 1×/day) data is duplicated here for ease of comparison. Error bars represent standard error.

## References

[acel12405-bib-0001] Anisimov VN , Zabezhinski MA , Popovich IG , Piskunova TS , Semenchenko AV , Tyndyk ML , Yurova MN , Antoch MP , Blagosklonny MV (2010) Rapamycin extends maximal lifespan in cancer‐prone mice. Am. J. Pathol. 176, 2092–2097. 20363920 10.2353/ajpath.2010.091050 PMC2861075

[acel12405-bib-0002] Anisimov VN , Zabezhinski MA , Popovich IG , Piskunova TS , Semenchenko AV , Tyndyk ML , Yurova MN , Rosenfeld SV , Blagosklonny MV (2011) Rapamycin increases lifespan and inhibits spontaneous tumorigenesis in inbred female mice. Cell Cycle 10, 4230–4236. 22107964 10.4161/cc.10.24.18486

[acel12405-bib-0003] Baker MD , Ezzati M , Aloisio GM , Tarnawa ED , Cuevas I , Nakada Y , Castrillon DH (2014) The small GTPase Rheb is required for spermatogenesis but not oogenesis. Reproduction 147, 615–625. 24713393 10.1530/REP-13-0304 PMC3982142

[acel12405-bib-0004] Barlow AD , Nicholson ML , Herbert TP (2013) Evidence for rapamycin toxicity in pancreatic beta‐cells and a review of the underlying molecular mechanisms. Diabetes 62, 2674–2682. 23881200 10.2337/db13-0106 PMC3717855

[acel12405-bib-0005] Byles V , Covarrubias AJ , Ben‐Sahra I , Lamming DW , Sabatini DM , Manning BD , Horng T (2013) The TSC‐mTOR pathway regulates macrophage polarization. Nat. Commun. 4, 2834. 24280772 10.1038/ncomms3834 PMC3876736

[acel12405-bib-0006] Festuccia WT , Pouliot P , Bakan I , Sabatini DM , Laplante M (2014) Myeloid‐specific Rictor deletion induces M1 macrophage polarization and potentiates *in vivo* pro‐inflammatory response to lipopolysaccharide. PLoS ONE 9, e95432. 24740015 10.1371/journal.pone.0095432 PMC3989321

[acel12405-bib-0007] Fontenot JD , Rasmussen JP , Williams LM , Dooley JL , Farr AG , Rudensky AY (2005) Regulatory T cell lineage specification by the forkhead transcription factor foxp3. Immunity 22, 329–341. 15780990 10.1016/j.immuni.2005.01.016

[acel12405-bib-0008] Formica RN Jr , Lorber KM , Friedman AL , Bia MJ , Lakkis F , Smith JD , Lorber MI (2004) The evolving experience using everolimus in clinical transplantation. Transplant. Proc. 36, 495S–499S. 15041395 10.1016/j.transproceed.2004.01.015

[acel12405-bib-0009] Goldberg EL , Smithey MJ , Lutes LK , Uhrlaub JL , Nikolich‐Zugich J (2014) Immune memory‐boosting dose of rapamycin impairs macrophage vesicle acidification and curtails glycolysis in effector CD8 cells, impairing defense against acute infections. J. Immunol. 193, 757–763. 24913978 10.4049/jimmunol.1400188 PMC4119820

[acel12405-bib-0010] Gu Y , Lindner J , Kumar A , Yuan W , Magnuson MA (2011) Rictor/mTORC2 is essential for maintaining a balance between beta‐cell proliferation and cell size. Diabetes 60, 827–837. 21266327 10.2337/db10-1194 PMC3046843

[acel12405-bib-0011] Hasty P , Livi CB , Dodds SG , Jones D , Strong R , Javors M , Fischer KE , Sloane L , Murthy K , Hubbard G , Sun L , Hurez V , Curiel TJ , Sharp ZD (2014) eRapa restores a normal life span in a FAP mouse model. Cancer Prev. Res. (Phila.) 7, 169–178. 24282255 10.1158/1940-6207.CAPR-13-0299 PMC4058993

[acel12405-bib-0012] Hinojosa CA , Mgbemena V , Van Roekel S , Austad SN , Miller RA , Bose S , Orihuela CJ (2012) Enteric‐delivered rapamycin enhances resistance of aged mice to pneumococcal pneumonia through reduced cellular senescence. Exp. Gerontol. 47, 958–965. 22981852 10.1016/j.exger.2012.08.013 PMC3490008

[acel12405-bib-0013] Houde VP , Brule S , Festuccia WT , Blanchard PG , Bellmann K , Deshaies Y , Marette A (2010) Chronic rapamycin treatment causes glucose intolerance and hyperlipidemia by upregulating hepatic gluconeogenesis and impairing lipid deposition in adipose tissue. Diabetes 59, 1338–1348. 20299475 10.2337/db09-1324 PMC2874694

[acel12405-bib-0014] Johnson SC , Rabinovitch PS , Kaeberlein M (2013) mTOR is a key modulator of ageing and age‐related disease. Nature 493, 338–345. 23325216 10.1038/nature11861 PMC3687363

[acel12405-bib-0015] Kimple ME , Keller MP , Rabaglia MR , Pasker RL , Neuman JC , Truchan NA , Brar HK , Attie AD (2013) Prostaglandin E2 receptor, EP3, is induced in diabetic islets and negatively regulates glucose‐ and hormone‐stimulated insulin secretion. Diabetes 62, 1904–1912. 23349487 10.2337/db12-0769 PMC3661627

[acel12405-bib-0016] Kumar A , Harris TE , Keller SR , Choi KM , Magnuson MA , Lawrence JC Jr (2008) Muscle‐specific deletion of rictor impairs insulin‐stimulated glucose transport and enhances Basal glycogen synthase activity. Mol. Cell. Biol. 28, 61–70. 17967879 10.1128/MCB.01405-07 PMC2223287

[acel12405-bib-0017] Kumar A , Lawrence JC Jr , Jung DY , Ko HJ , Keller SR , Kim JK , Magnuson MA , Harris TE (2010) Fat cell‐specific ablation of rictor in mice impairs insulin‐regulated fat cell and whole‐body glucose and lipid metabolism. Diabetes 59, 1397–1406. 20332342 10.2337/db09-1061 PMC2874700

[acel12405-bib-0018] Lamming DW , Ye L , Katajisto P , Goncalves MD , Saitoh M , Stevens DM , Davis JG , Salmon AB , Richardson A , Ahima RS , Guertin DA , Sabatini DM , Baur JA (2012) Rapamycin‐induced insulin resistance is mediated by mTORC2 loss and uncoupled from longevity. Science 335, 1638–1643. 22461615 10.1126/science.1215135 PMC3324089

[acel12405-bib-0019] Lamming DW , Ye L , Astle CM , Baur JA , Sabatini DM , Harrison DE (2013a) Young and old genetically heterogeneous HET3 mice on a rapamycin diet are glucose intolerant but insulin sensitive. Aging Cell 12, 712–718. 23648089 10.1111/acel.12097 PMC3727050

[acel12405-bib-0020] Lamming DW , Ye L , Sabatini DM , Baur JA (2013b) Rapalogs and mTOR inhibitors as anti‐aging therapeutics. J. Clin. Invest. 123, 980–989. 23454761 10.1172/JCI64099 PMC3582126

[acel12405-bib-0021] Lamming DW , Demirkan G , Boylan JM , Mihaylova MM , Peng T , Ferreira J , Neretti N , Salomon A , Sabatini DM , Gruppuso PA (2014a) Hepatic signaling by the mechanistic target of rapamycin complex 2 (mTORC2). FASEB J. 28, 300–315. 24072782 10.1096/fj.13-237743 PMC3868844

[acel12405-bib-0022] Lamming DW , Mihaylova MM , Katajisto P , Baar EL , Yilmaz OH , Hutchins A , Gultekin Y , Gaither R , Sabatini DM (2014b) Depletion of Rictor, an essential protein component of mTORC2, decreases male lifespan. Aging Cell 13, 911–917. 25059582 10.1111/acel.12256 PMC4172536

[acel12405-bib-0023] Lee K , Gudapati P , Dragovic S , Spencer C , Joyce S , Killeen N , Magnuson MA , Boothby M (2010) Mammalian target of rapamycin protein complex 2 regulates differentiation of Th1 and Th2 cell subsets via distinct signaling pathways. Immunity 32, 743–753. 20620941 10.1016/j.immuni.2010.06.002 PMC2911434

[acel12405-bib-0024] Leontieva OV , Paszkiewicz GM , Blagosklonny MV (2014) Weekly administration of rapamycin improves survival and biomarkers in obese male mice on high‐fat diet. Aging Cell 13, 616–622. 24655348 10.1111/acel.12211 PMC4326934

[acel12405-bib-0025] Levy JC , Matthews DR , Hermans MP (1998) Correct homeostasis model assessment (HOMA) evaluation uses the computer program. Diabetes Care 21, 2191–2192. 9839117 10.2337/diacare.21.12.2191

[acel12405-bib-0026] Liu Y , Diaz V , Fernandez E , Strong R , Ye L , Baur JA , Lamming DW , Richardson A , Salmon AB (2014) Rapamycin‐induced metabolic defects are reversible in both lean and obese mice. Aging (Albany NY) 6, 742–754. 25324470 10.18632/aging.100688 PMC4221917

[acel12405-bib-0027] Mahe E , Morelon E , Lechaton S , Sang KH , Mansouri R , Ducasse MF , Mamzer‐Bruneel MF , de Prost Y , Kreis H , Bodemer C (2005) Cutaneous adverse events in renal transplant recipients receiving sirolimus‐based therapy. Transplantation 79, 476–482. 15729175 10.1097/01.tp.0000151630.25127.3a

[acel12405-bib-0028] Makki K , Taront S , Molendi‐Coste O , Bouchaert E , Neve B , Eury E , Lobbens S , Labalette M , Duez H , Staels B , Dombrowicz D , Froguel P , Wolowczuk I (2014) Beneficial metabolic effects of rapamycin are associated with enhanced regulatory cells in diet‐induced obese mice. PLoS ONE 9, e92684. 24710396 10.1371/journal.pone.0092684 PMC3977858

[acel12405-bib-0029] Mannick JB , Del Giudice G , Lattanzi M , Valiante NM , Praestgaard J , Huang B , Lonetto MA , Maecker HT , Kovarik J , Carson S , Glass DJ , Klickstein LB (2014) mTOR inhibition improves immune function in the elderly. Sci. Transl. Med. 6, 268ra179. 10.1126/scitranslmed.3009892 25540326

[acel12405-bib-0030] Mather K (2009) Surrogate measures of insulin resistance: of rats, mice, and men. Am. J. Physiol. Endocrinol. Metab. 296, E398–E399. 19171846 10.1152/ajpendo.90889.2008

[acel12405-bib-0031] Moller AP (1989) Ejaculate quality, testes size and sperm production in mammals. Funct. Ecol. 3, 91–96.

[acel12405-bib-0032] Powell JD , Pollizzi KN , Heikamp EB , Horton MR (2012) Regulation of immune responses by mTOR. Annu. Rev. Immunol. 30, 39–68. 22136167 10.1146/annurev-immunol-020711-075024 PMC3616892

[acel12405-bib-0033] Sankhala K , Mita A , Kelly K , Mahalingam D , Giles F , Mita M (2009) The emerging safety profile of mTOR inhibitors, a novel class of anticancer agents. Target. Oncol. 4, 135–142. 19381454 10.1007/s11523-009-0107-z

[acel12405-bib-0034] Sarbassov DD , Ali SM , Sengupta S , Sheen JH , Hsu PP , Bagley AF , Markhard AL , Sabatini DM (2006) Prolonged rapamycin treatment inhibits mTORC2 assembly and Akt/PKB. Mol. Cell 22, 159–168. 16603397 10.1016/j.molcel.2006.03.029

[acel12405-bib-0035] Schreiber KH , Ortiz D , Academia EC , Anies AC , Liao CY , Kennedy BK (2015) Rapamycin‐mediated mTORC2 inhibition is determined by the relative expression of FK506‐binding proteins. Aging Cell 14, 265–273. 25652038 10.1111/acel.12313 PMC4364838

[acel12405-bib-0036] Selman C , Tullet JM , Wieser D , Irvine E , Lingard SJ , Choudhury AI , Claret M , Al‐Qassab H , Carmignac D , Ramadani F , Woods A , Robinson IC , Schuster E , Batterham RL , Kozma SC , Thomas G , Carling D , Okkenhaug K , Thornton JM , Partridge L , Gems D , Withers DJ (2009) Ribosomal protein S6 kinase 1 signaling regulates mammalian life span. Science 326, 140–144. 19797661 10.1126/science.1177221 PMC4954603

[acel12405-bib-0037] Tang XL , Smith TR , Kumar V (2005) Specific control of immunity by regulatory CD8 T cells. Cell. Mol. Immunol. 2, 11–19. 16212906

[acel12405-bib-0038] Turner AP , Shaffer VO , Araki K , Martens C , Turner PL , Gangappa S , Ford ML , Ahmed R , Kirk AD , Larsen CP (2011) Sirolimus enhances the magnitude and quality of viral‐specific CD8 + T‐cell responses to vaccinia virus vaccination in rhesus macaques. Am. J. Transplant. 11, 613–618. 21342450 10.1111/j.1600-6143.2010.03407.x PMC3076606

[acel12405-bib-0039] Wilkinson JE , Burmeister L , Brooks SV , Chan CC , Friedline S , Harrison DE , Hejtmancik JF , Nadon N , Strong R , Wood LK , Woodward MA , Miller RA (2012) Rapamycin slows aging in mice. Aging Cell 11, 675–682. 22587563 10.1111/j.1474-9726.2012.00832.x PMC3434687

[acel12405-bib-0040] Wu JJ , Liu J , Chen EB , Wang JJ , Cao L , Narayan N , Fergusson MM , Rovira II , Allen M , Springer DA , Lago CU , Zhang S , DuBois W , Ward T , deCabo R , Gavrilova O , Mock B , Finkel T (2013) Increased mammalian lifespan and a segmental and tissue‐specific slowing of aging after genetic reduction of mTOR expression. Cell Rep. 4, 913–920. 23994476 10.1016/j.celrep.2013.07.030 PMC3784301

[acel12405-bib-0041] Yang SB , Lee HY , Young DM , Tien AC , Rowson‐Baldwin A , Shu YY , Jan YN , Jan LY (2012) Rapamycin induces glucose intolerance in mice by reducing islet mass, insulin content, and insulin sensitivity. J. Mol. Med. (Berl) 90, 575–585. 22105852 10.1007/s00109-011-0834-3 PMC3354320

[acel12405-bib-0042] Zelenay S , Lopes‐Carvalho T , Caramalho I , Moraes‐Fontes MF , Rebelo M , Demengeot J (2005) Foxp3 + CD25‐ CD4 T cells constitute a reservoir of committed regulatory cells that regain CD25 expression upon homeostatic expansion. Proc. Natl Acad. Sci. USA 102, 4091–4096. 15753306 10.1073/pnas.0408679102 PMC554795

