# Supplementary figures and images for: Alternative rapamycin treatment regimens mitigate the impact of rapamycin on glucose homeostasis and the immune system
Source: Aging Cell. 2015 Oct 13;15(1):28–38. doi: 10.1111/acel.12405 (PMC4717280; doi:10.1111/acel.12405)

Figure S1

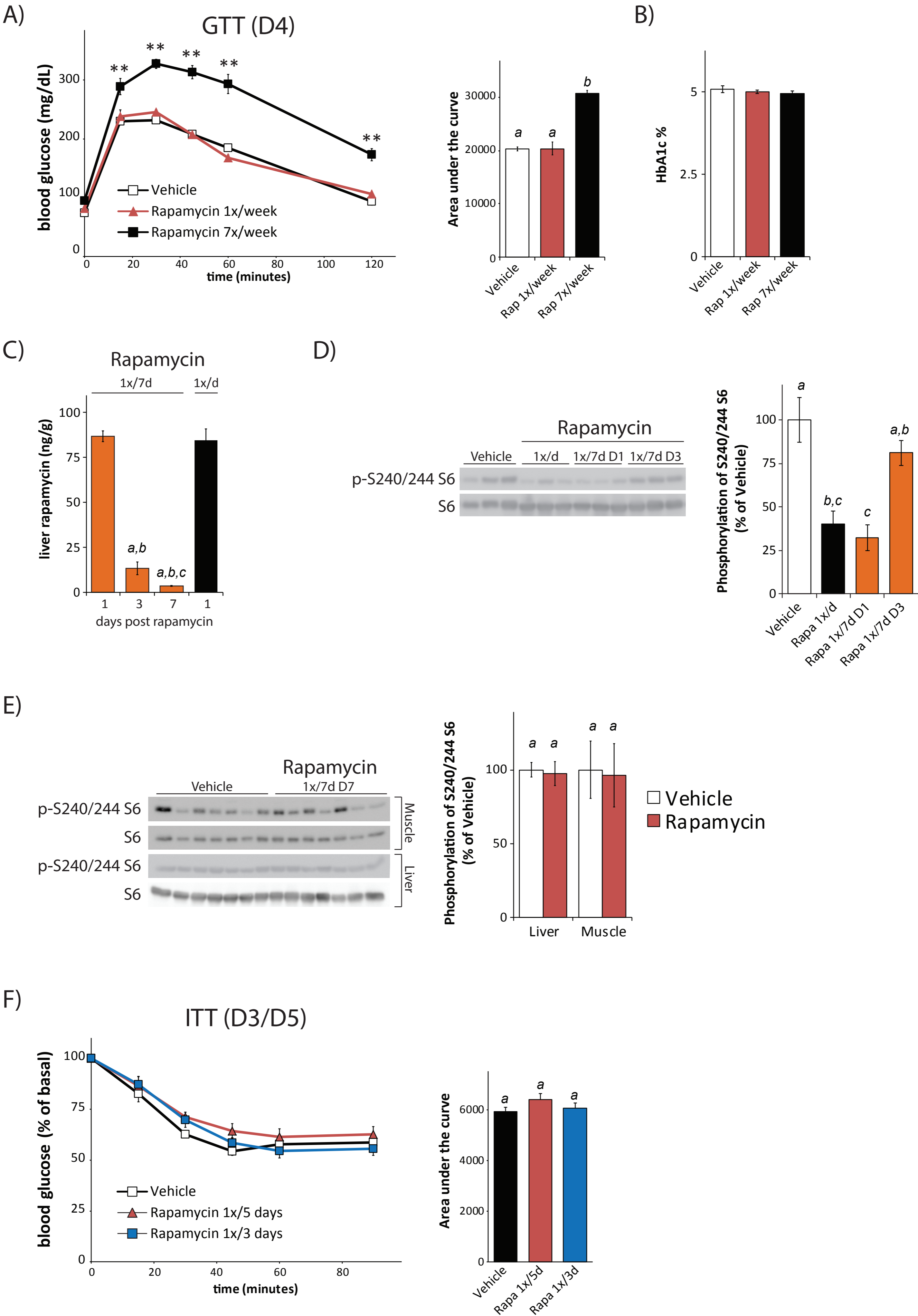

Supplement: Supplementary file 1 — Figure S1 Impact of weekly rapamycin on glucose homeostasis and mTORC1 activity in the liver. A) Glucose tolerance test on male C57BL/6J mice treated with vehicle or with 2 mg/kg rapamycin (1×/day or 1×/7 days) for 5 weeks, tested 4 days (D4) after the most recent injection of the Rapamycin 1×/7 days group [n = 10 vehicle, n = 9 Rapamycin 1×/day, and n = 11 Rapamycin 1×/7 days rapamycin; for GTT, *P < 0.05, **P < 0.0001 vs. all groups, Tukey‐Kramer test following two‐way repeated‐measures anova; for AUC, means with the same letter are not significantly different from each other (Tukey–Kramer test following one‐way anova, p < 0.05)]. B) HbA1c was assayed from the whole blood of mice treated with vehicle or with 2 mg/kg rapamycin (1×/day or 1×/7 days). C) Rapamycin concentration in liver from male C57Bl/6J mice treated with 2 mg/kg rapamycin (1×/day or 1×/7 days) for 8 weeks; blood from 1×/7 days mice was collected 1 day (D1), 3 days (D3) or 7 days (D7) after the more recent rapamycin injection (n = 3‐6/group; a = P < 0.05 vs. 1×/7 days D1; b = P < 0.05 vs. 1×/day daily rapamycin mice; c = P < 0.05 vs. 1×/7 days D3; two‐tailed t‐test). (D) Western blotting analysis and quantification of phosphorylated S6 (Ser 240/244) in liver [n = 9 vehicle, 7 1×/day rapamycin, 3 rapamycin 1×/7days D1, 6 rapamycin 1×/7days D3; means with the same letter are not significantly different from each other (Tukey–Kramer test following one‐way anova, P < 0.05)]. (E) Western blotting analysis and quantification of phosphorylated S6 (Ser 240/244) in liver and muscle [n = 7 per group, means with the same letter are not significantly different from each other (Tukey–Kramer test following one‐way anova, P < 0.05)]. F) Insulin tolerance test on mice treated intermittently with either vehicle or with 2 mg/kg rapamycin (1×/3 or 5 days) for 2 weeks [n = 10 mice/group, * = P < 0.05, Tukey‐Kramer test following two‐way repeated‐measures anova; for AUC, means with the same letter are not significantly [file ACEL-15-028-s001.pdf]

Figure S2

A)

Liver

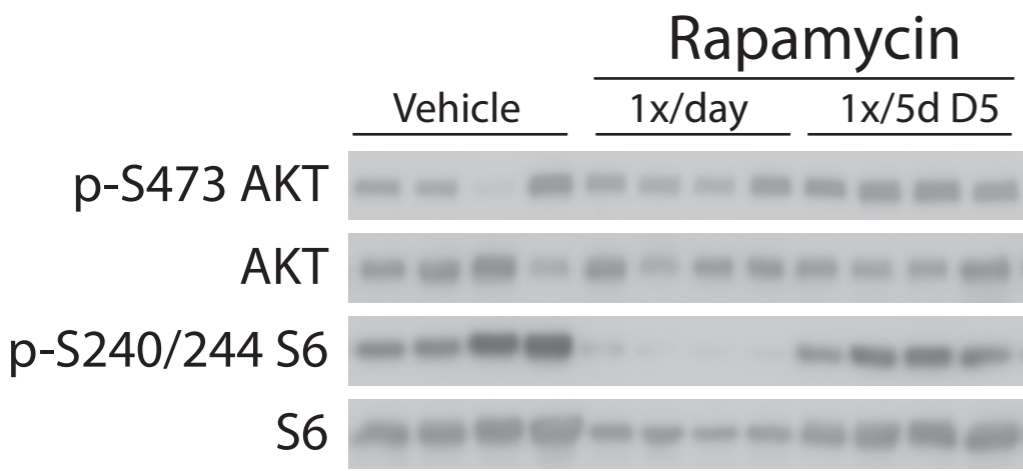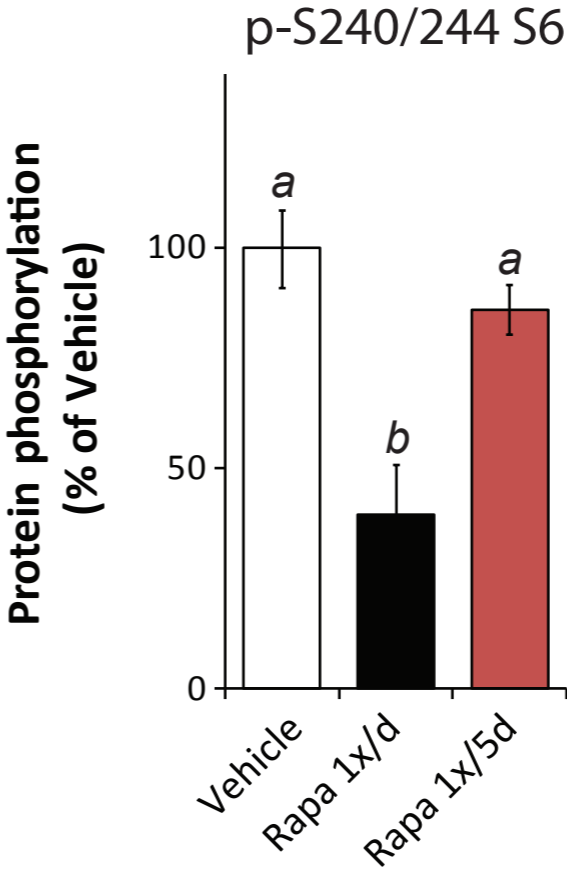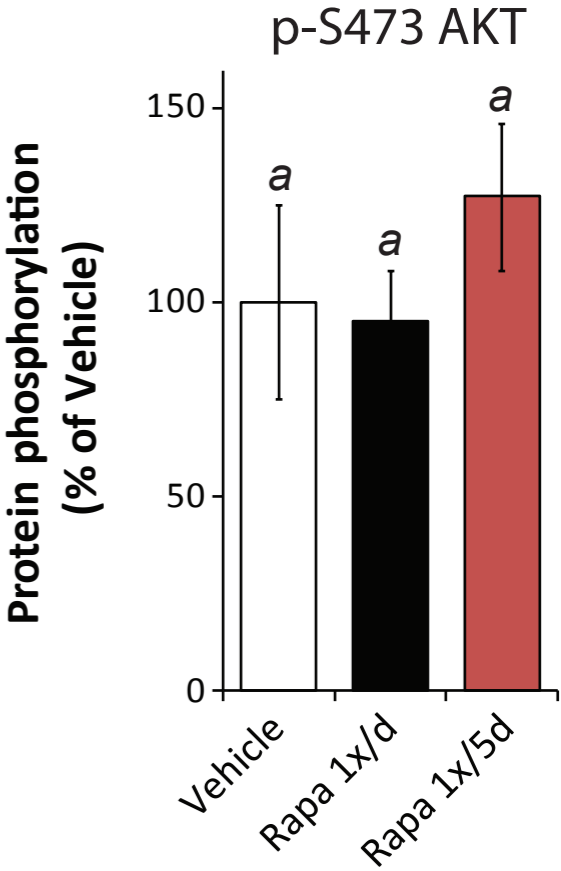

B)

Heart

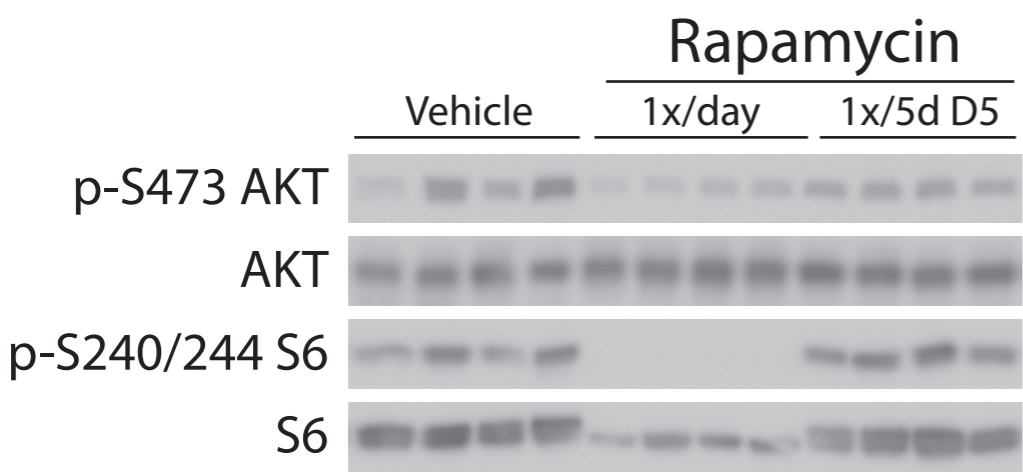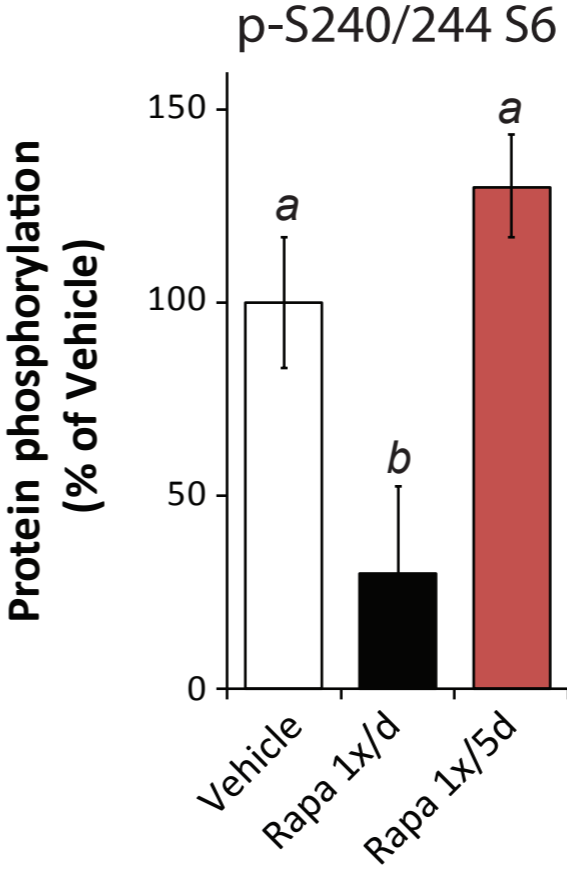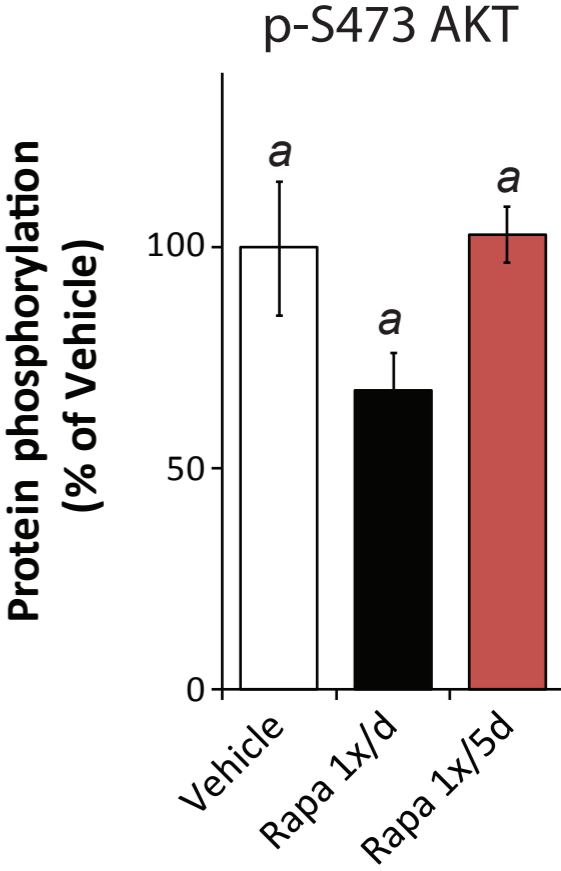

C)

Islets

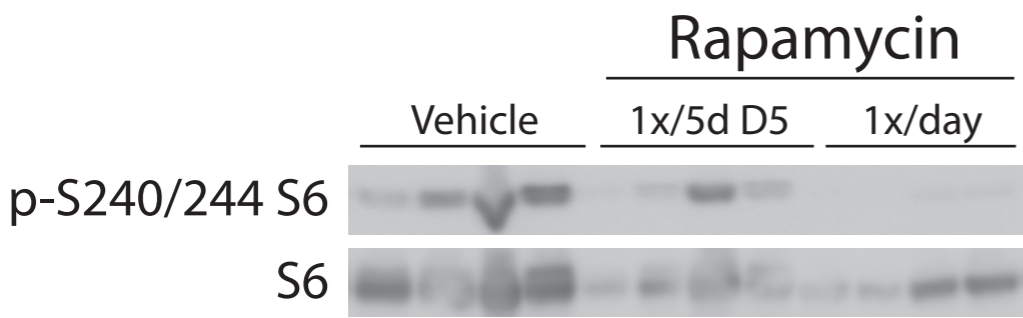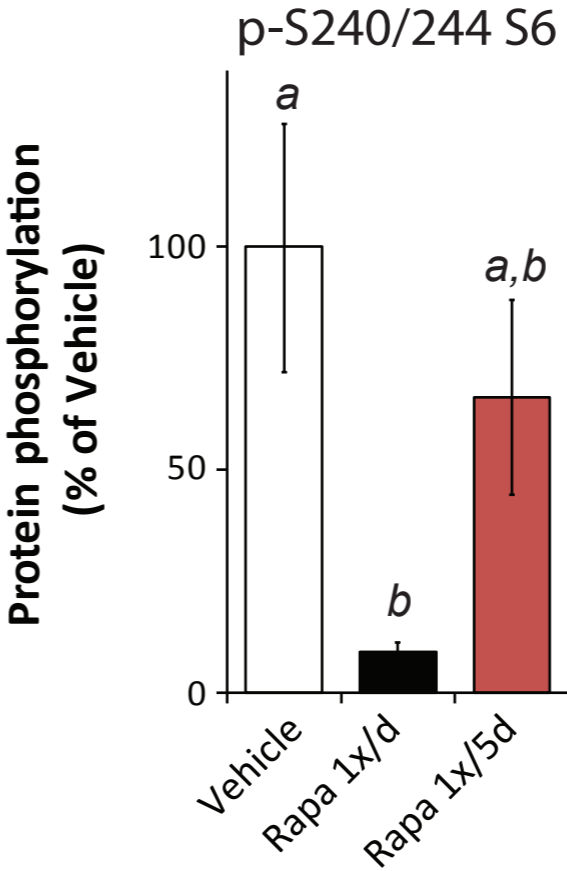

Supplement: Supplementary file 2 — Figure S2 Impact of intermittent rapamycin on liver and heart. A) Liver lysate, B) Heart tissue lysate and C) Pancreatic islet lysate was analyzed by western blotting and the phosphorylation of S6 240/244 and AKT S473 relative to their respective total protein was quantified. Tissues were collected from mice treated with vehicle or rapamycin (1×/day or 1×/5 days) for 8 weeks, with the tissue collection scheduled such that the intermittent rapamycin treatment group was sacrificed 5 days after the previous rapamycin injection. Mice were fasted overnight and sacrificed following stimulation with 0.75 U/kg insulin for 15 min. Islets were isolated as described prior to tissue collection [n = 4–9/group, means with the same letter are not significantly different from each other (Tukey–Kramer test following one‐way anova, P < 0.05)]. Error bars represent standard error. [file ACEL-15-028-s002.pdf]

Figure S3

A)

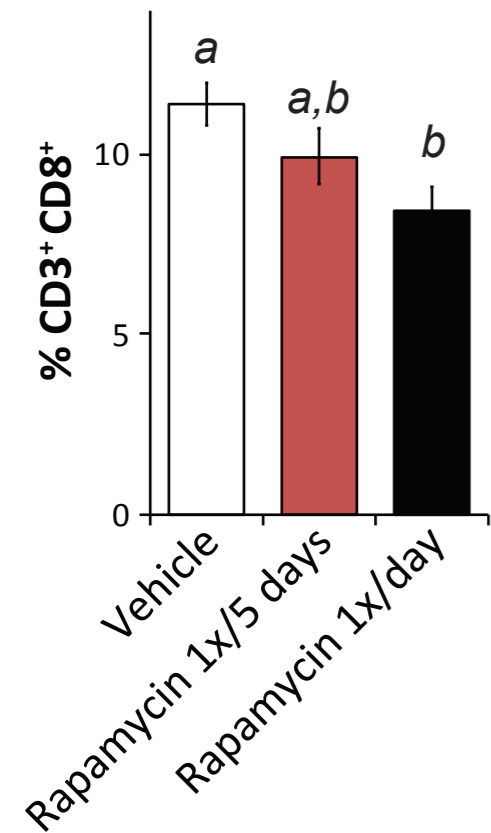

B)

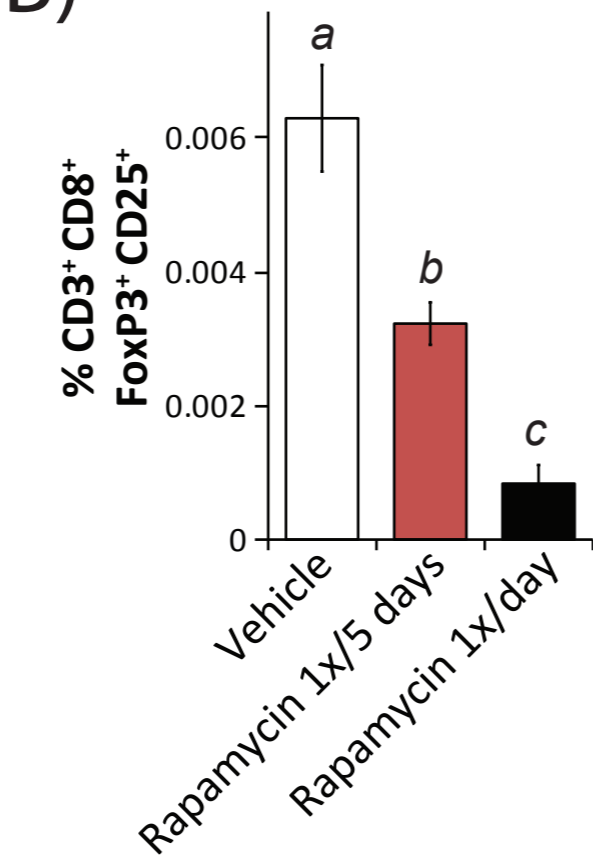

C)

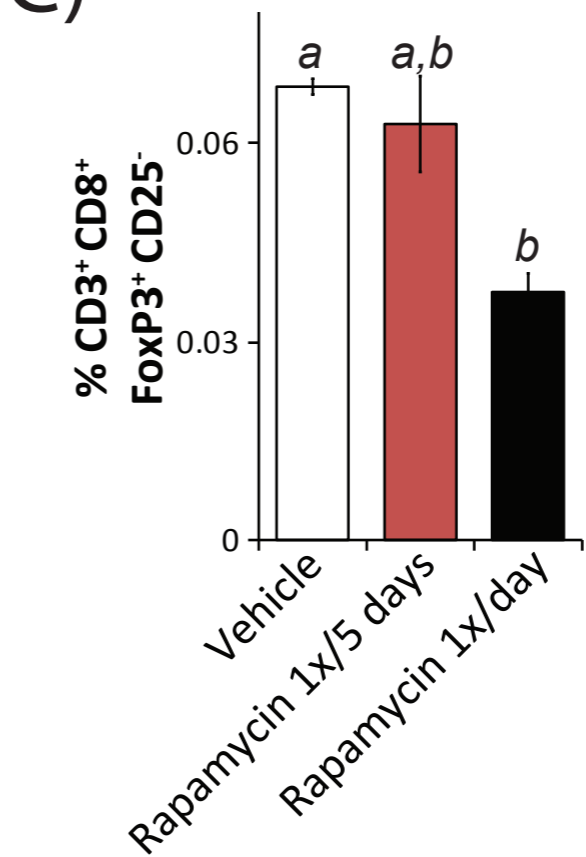

Supplement: Supplementary file 3 — Figure S3 Intermittent rapamycin treatment has a reduced but significant impact on the immune system. A‐H) Flow cytometry analysis (expressed as percent of total live cells) of CD8+ splenocytes from male C57BL/6J mice treated with vehicle or rapamycin (1×/day or 1×/5 days) for 8 weeks (n = 6–8 mice/group, means with the same letter are not significantly different from each other (Tukey–Kramer test following one‐way anova, P < 0.05). Error bars represent standard error. [file ACEL-15-028-s003.pdf]

Figure S4

A)

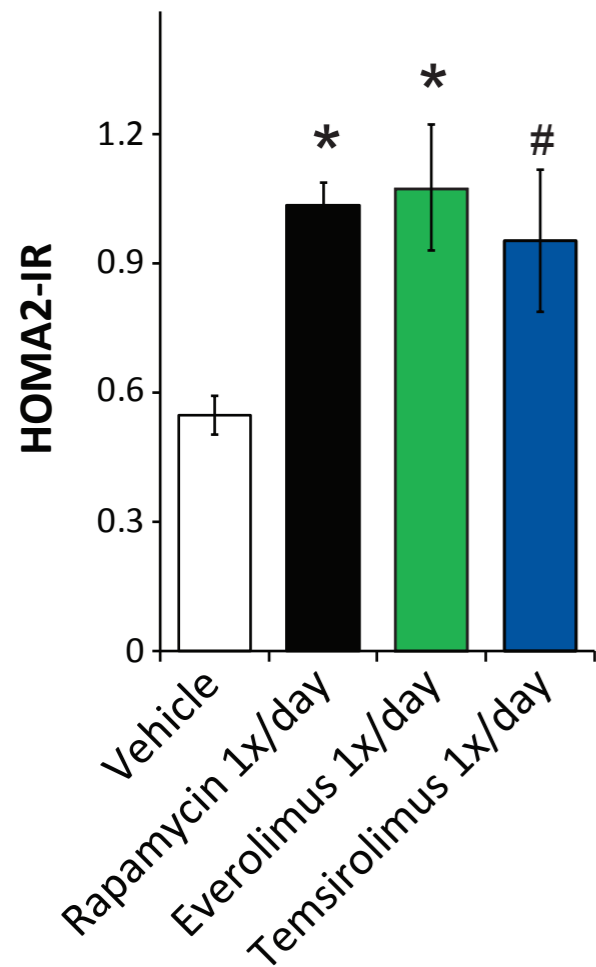

B)

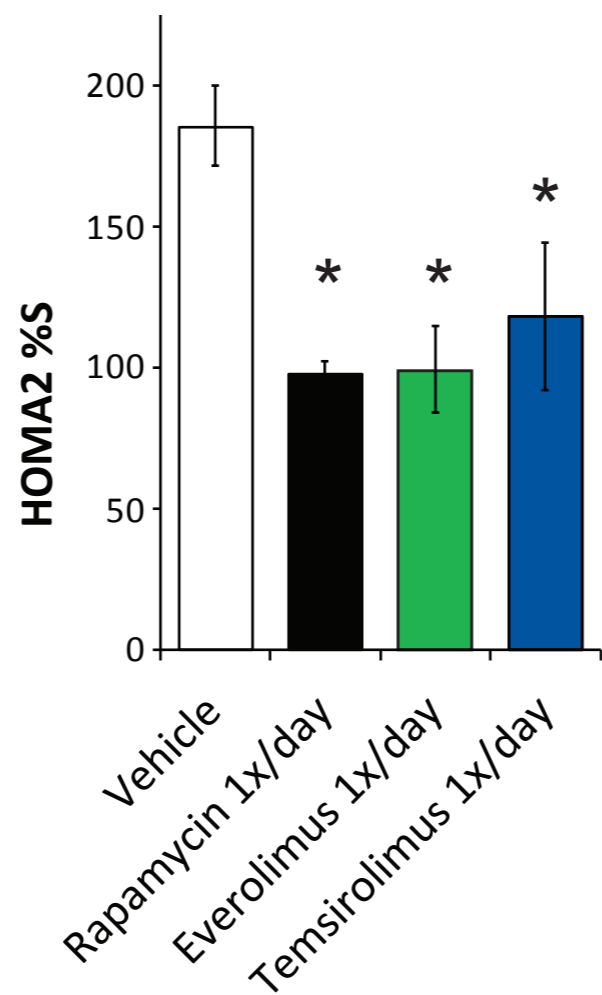

C)

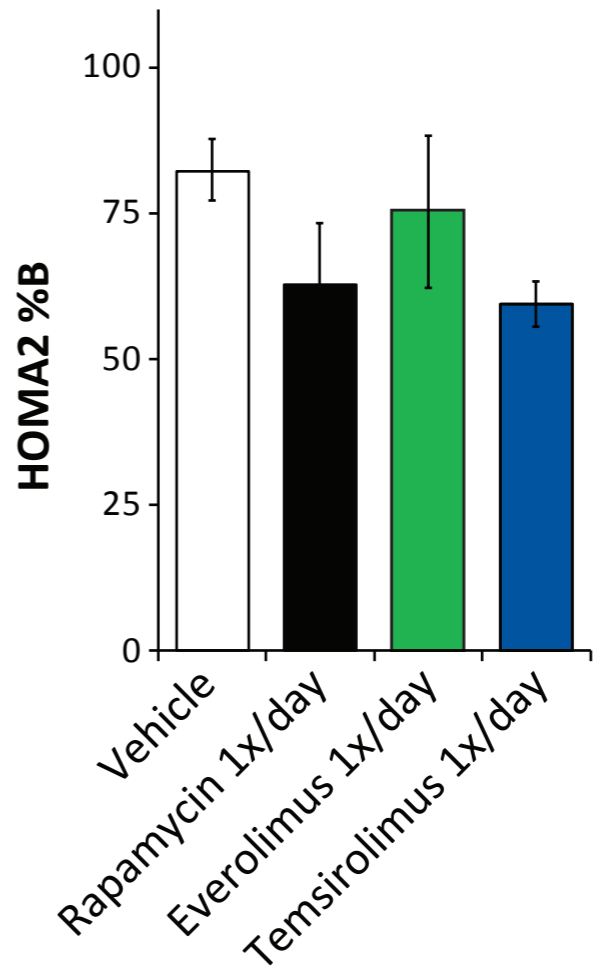

Supplement: Supplementary file 4 — Figure S4 Impact of rapalogs on insulin resistance. A‐C) HOMA2‐IR and HOMA2%B was calculated using the fasting insulin data in Figure 5C and fasting glucose data from the same mice (n = 4/group, # = P ≤ 0.08 vs. vehicle, * = P ≤ 0.05 vs. vehicle, Dunnett's test following one‐way anova). The experiments presented here were conducted in parallel with the experiment presented in Figure 2, and the vehicle and daily (Rapamycin 1×/day) data is duplicated here for ease of comparison. Error bars represent standard error. [file ACEL-15-028-s004.pdf]

Figure S5

A)

Liver

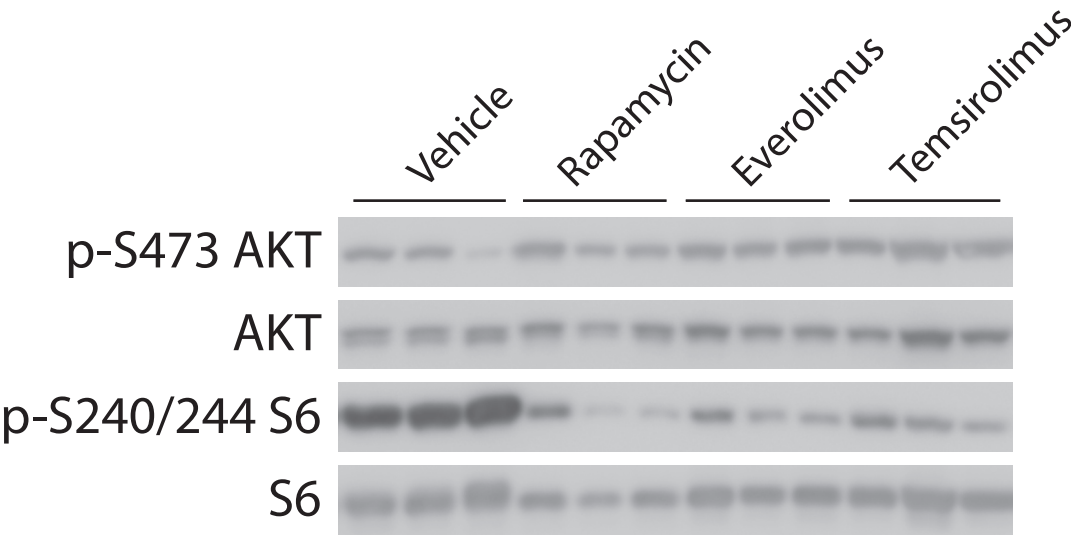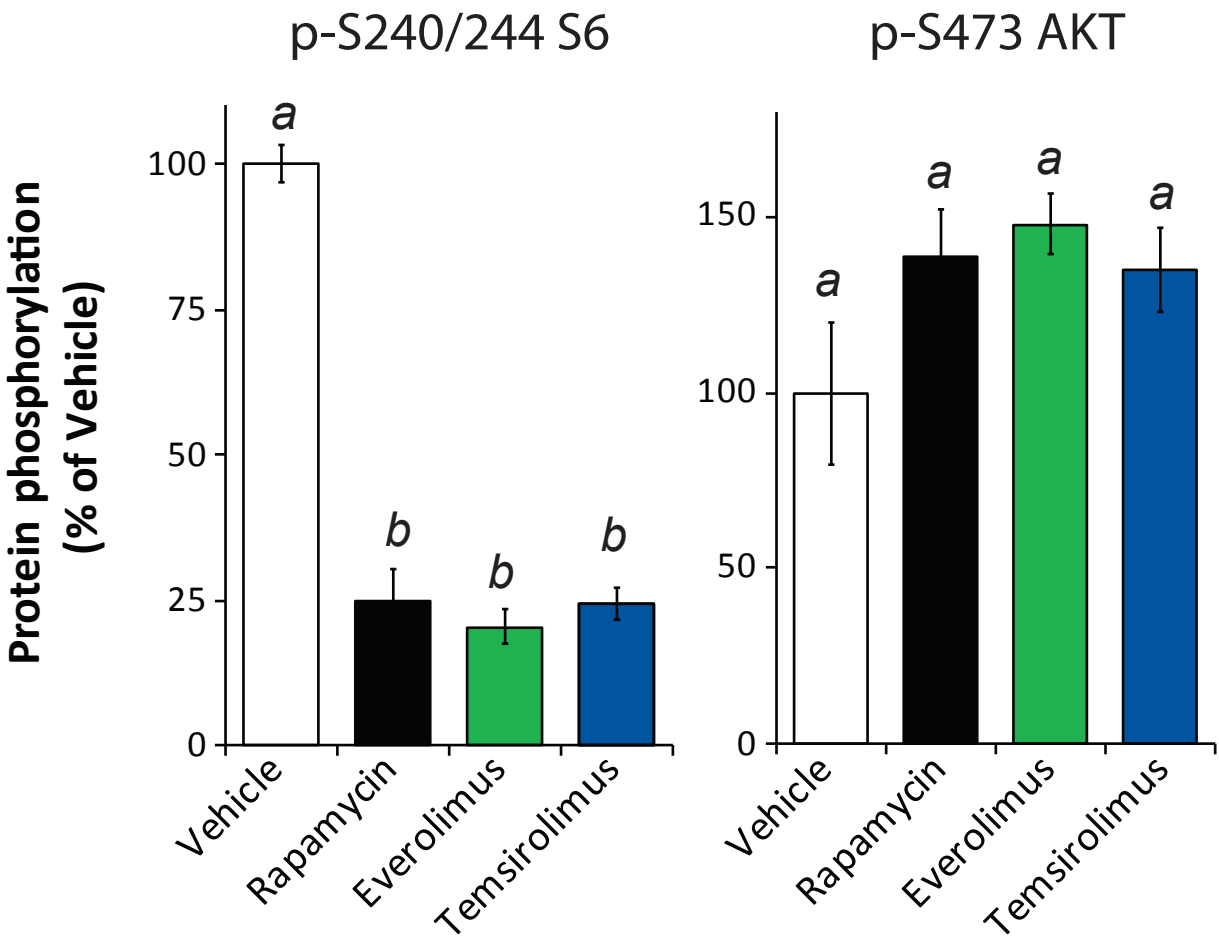

B)

Adipose

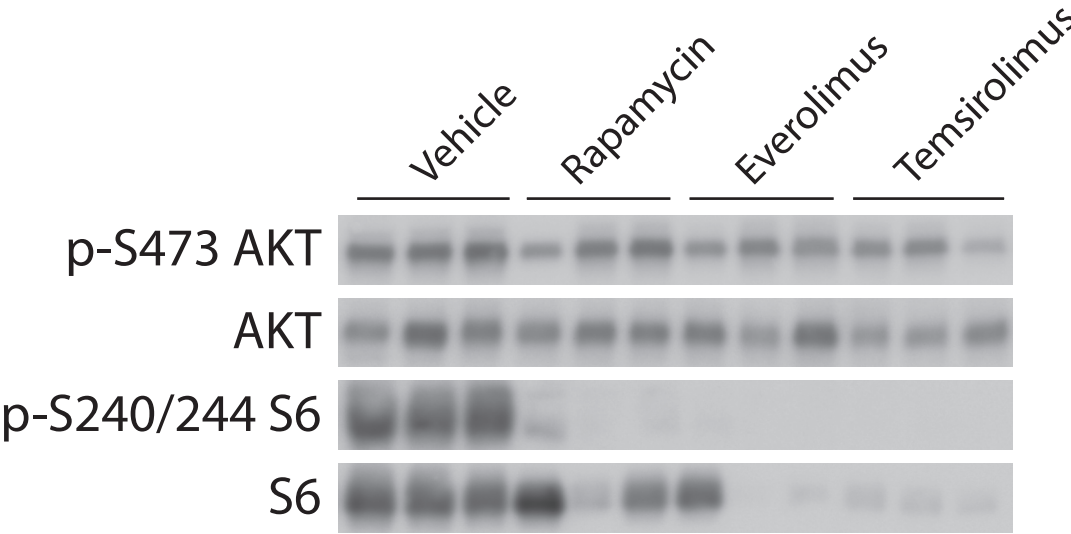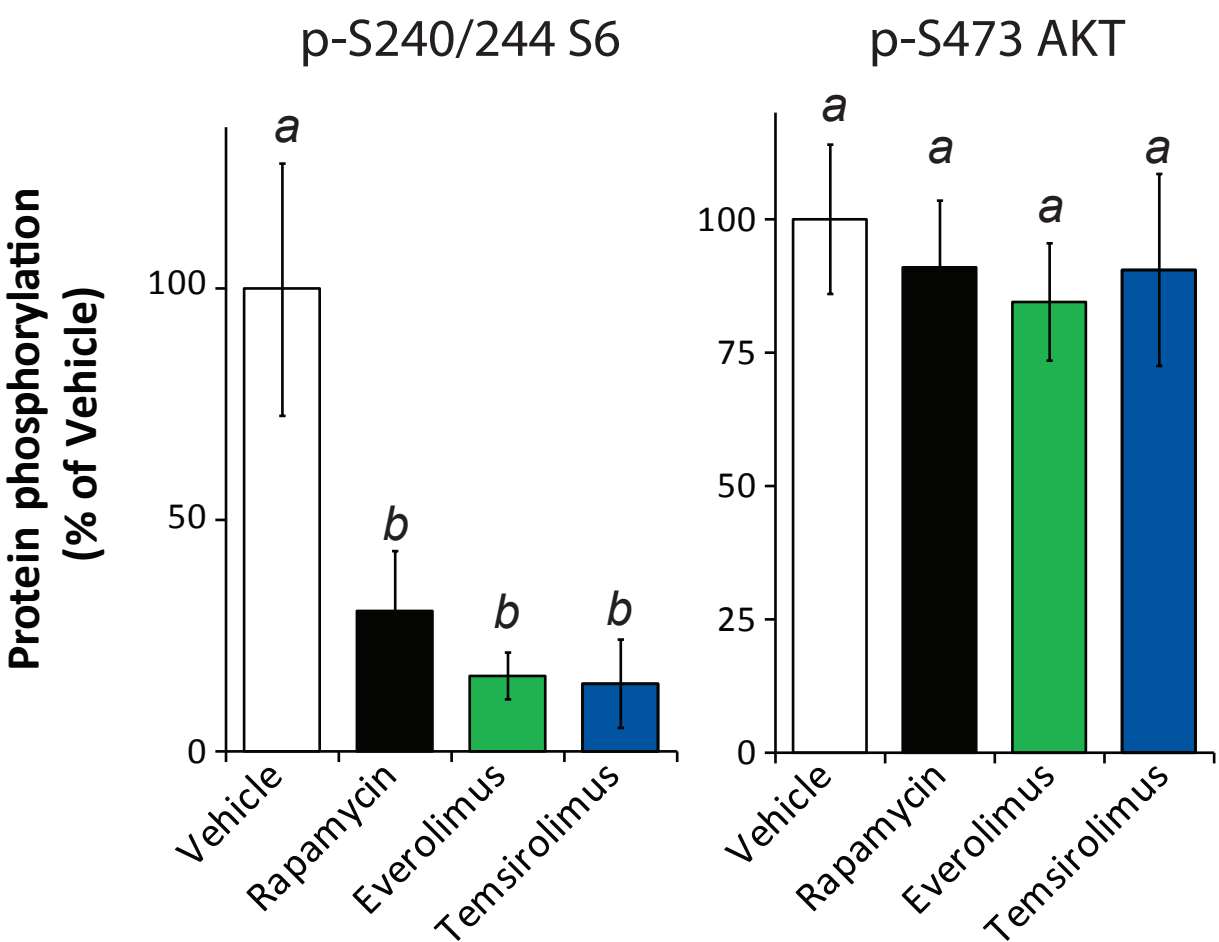

C)

Heart

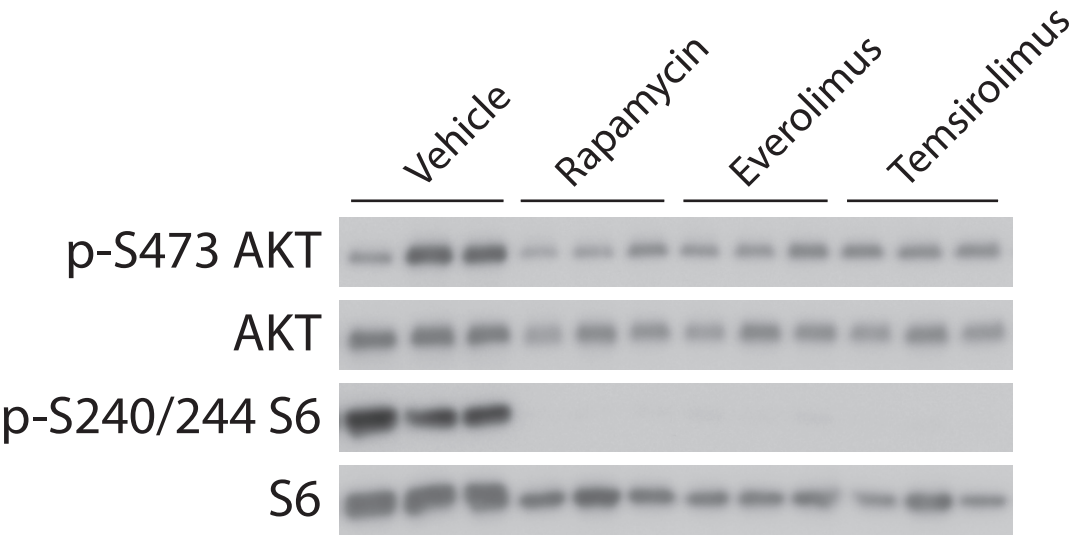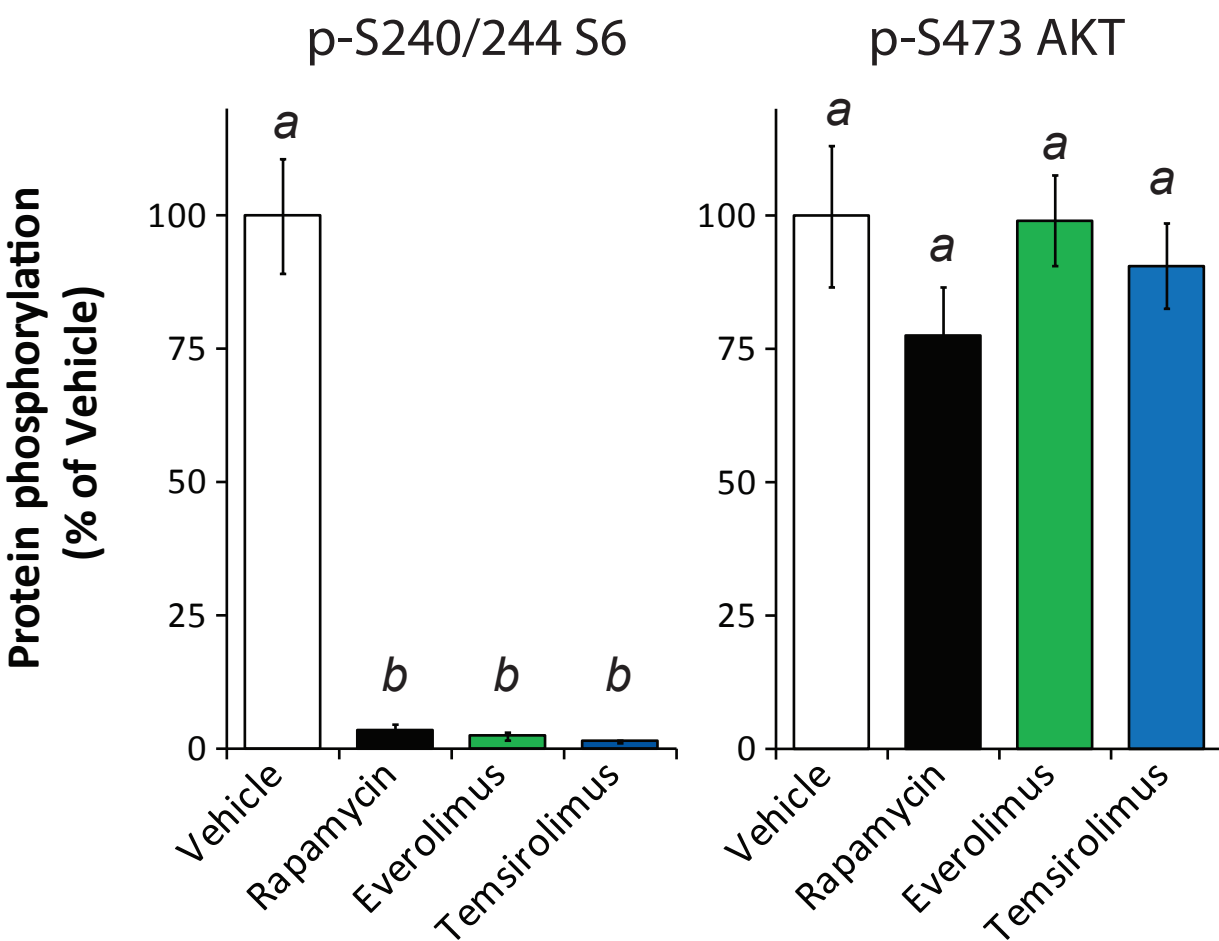

Supplement: Supplementary file 5 — Figure S5 Impact of rapamycin analogs on mTOR signaling in liver, adipose and heart. A‐C) Liver, adipose and heart lysate was analyzed by western blotting and the phosphorylation of S6 240/244 and AKT S473 relative to their respective total protein was quantified [n = 4–9/group, means with the same letter are not significantly different from each other (Tukey–Kramer test following one‐way anova, P < 0.05)]. Error bars represent standard error. [file ACEL-15-028-s005.pdf]

Figure S6

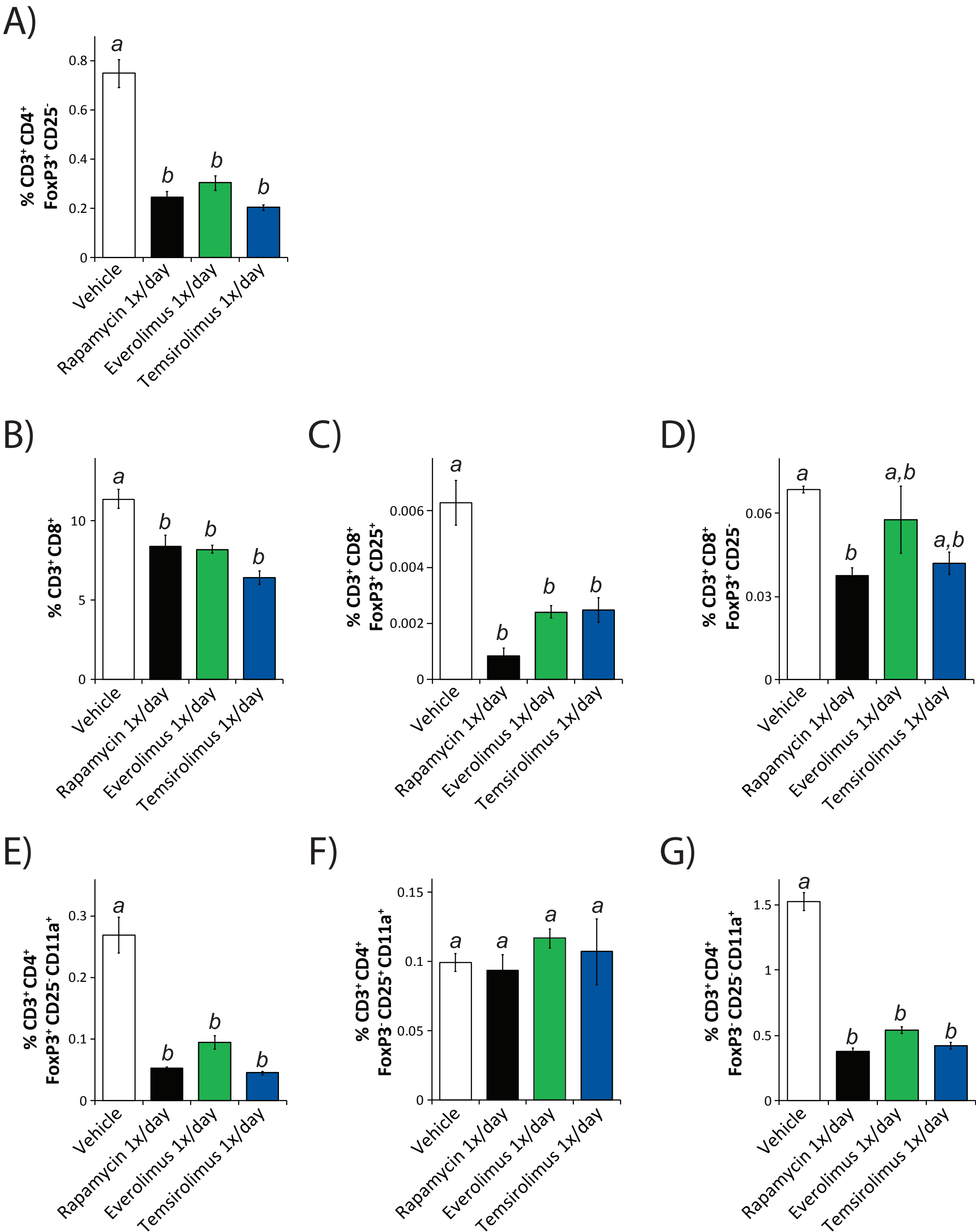

Supplement: Supplementary file 6 — Figure S6 Impact of rapamycin analogs on splenocyte populations. Flow cytometry analysis (expressed as percent of total live cells) on splenocytes isolated from each treatment group [n = 3–8 mice/group, means with the same letter are not significantly different from each other (Tukey–Kramer test following one‐way anova, P < 0.05)]. The experiments presented here were conducted in parallel with the experiment presented in Figures 4 and S3, and the vehicle and daily (Rapamycin 1×/day) data is duplicated here for ease of comparison. Error bars represent standard error. [file ACEL-15-028-s006.pdf]
