# Supplementary material for: Alternative rapamycin treatment regimens mitigate the impact of rapamycin on glucose homeostasis and the immune system
Source: Aging Cell. 2015 Oct 13;15(1):28–38. doi: 10.1111/acel.12405 (PMC4717280; doi:10.1111/acel.12405)
Supplement: Supplementary file 7 [file ACEL-15-028-s007.docx]

**Supplemental methods**

**Rapamycin quantification**

Rapamycin quantification was performed by the Bioanalytical Pharmacology Core Facility of University of Texas Health Science Center San Antonio Nathan Shock Center. Quantification of rapamycin levels in blood and liver was by tandem HPLC-MS as previously described ([Livi *et al.* 2013](#_ENREF_1)).

**Islet isolation and *ex vivo* glucose stimulated insulin secretion (GSIS) assay**

Islets were isolated as previously described ([Neuman *et al.* 2014](#_ENREF_2)). Islets were then immediately transferred to a 96-well v-bottom plate with RPMI 1640 medium with 10% FBS, penicillin/streptomycin, and 11.1 mM glucose for a high-throughput GSIS assay. Briefly, following a 48-hour incubation period, islets were treated with a low glucose (1.7mM) Krebs-Ringer bicarbonate buffer pre-incubation solution for 45-minutes followed by stimulatory high glucose (16.7mM) Krebs-Ringer bicarbonate buffer solution for 45-minutes. Secretory media was then saved and islets were lysed with a Cell Signaling Technologies lysis buffer (9803). Insulin secretion and content were analyzed by ELISA ([Rabaglia *et al.* 2005](#_ENREF_3)).

**Splenocyte preparation and flow cytometry**

Splenocytes were prepared following a procedure from Life Technologies. Briefly, spleens were collected in a buffer containing PBS, 0.1% BSA, and 0.6% Na-citrate, and then macerated through a 70μM filter using a syringe plunger. Following a centrifugation at 300 x g for 10 minutes, cells were resuspended in the same buffer, and recentrifuged. The splenocytes were suspended in PBS and 0.1% BSA with Ca^2+^ and Mg^2+^, and incubated with approximately 100 units of DNAase. Splenocytes were then filtered through a 40μM filter and red blood cells were lysed. Splenocytes were then centrifuged, suspended in PBS with 0.1% BSA, and brought to the UWCCC Flow Cytometry Lab for immunostaining and flow cytometry on a BD LSRII (San Jose, California). Data was collected using BD FACSDiva, Version 8.0 and analyzed with FlowJo X, Version 10.0.7r2 (FlowJo, LLC, Ashland, OR).

**Supplemental References**

Livi CB, Hardman RL, Christy BA, Dodds SG, Jones D, Williams C, Strong R, Bokov A, Javors MA, Ikeno Y, Hubbard G, Hasty P, Sharp ZD (2013). Rapamycin extends life span of Rb1+/- mice by inhibiting neuroendocrine tumors. *Aging (Albany NY)*. **5**, 100-110.

Neuman JC, Truchan NA, Joseph JW, Kimple ME (2014). A method for mouse pancreatic islet isolation and intracellular cAMP determination. *Journal of visualized experiments : JoVE*, e50374.

Rabaglia ME, Gray-Keller MP, Frey BL, Shortreed MR, Smith LM, Attie AD (2005). Alpha-Ketoisocaproate-induced hypersecretion of insulin by islets from diabetes-susceptible mice. *Am J Physiol Endocrinol Metab*. **289**, E218-224.
